# Predictive value of MRI-based deep learning model for lymphovascular invasion status in node-negative invasive breast cancer

**DOI:** 10.1038/s41598-024-67217-0

**Published:** 2024-07-13

**Authors:** Rong Liang, Fangfang Li, Jingyuan Yao, Fang Tong, Minghui Hua, Junjun Liu, Chenlei Shi, Lewen Sui, Hong Lu

**Affiliations:** 1 Department of Breast Imaging, Tianjin Medical University Cancer Institute and Hospital, National Clinical Research Center for Cancer, West Huan-Hu Road, Ti Yuan Bei, Hexi District, Tianjin, 300060 People’s Republic of China; 2 Key Laboratory of Cancer Prevention and Therapy, Tianjin’s Clinical Research Center for Cancer, Key Laboratory of Breast Cancer Prevention and Therapy, Tianjin Medical University, Ministry of Education, Tianjin, People’s Republic of China; 3 Department of Physiology and Biochemistry, School of Fundamental Medicine, Shanghai University of Medicine and Health Sciences, Shanghai, People’s Republic of China; 4 Institute of Wound Prevention and Treatment, Shanghai University of Medicine and Health Sciences, Shanghai, People’s Republic of China; 5 Shanghai University of Medicine and Health Sciences Affiliated Zhoupu Hospital, Shanghai, People’s Republic of China; 6 Department of Radiology, Chest Hospital, Tianjin University, Tianjin, People’s Republic of China

**Keywords:** Breast cancer, Lymphovascular invasion, Magnetic resonance imaging, Deep learning, Breast cancer, Cancer imaging

## Abstract

To retrospectively assess the effectiveness of deep learning (DL) model, based on breast magnetic resonance imaging (MRI), in predicting preoperative lymphovascular invasion (LVI) status in patients diagnosed with invasive breast cancer who have negative axillary lymph nodes (LNs). Data was gathered from 280 patients, including 148 with LVI-positive and 141 with LVI-negative lesions. These patients had undergone preoperative breast MRI and were histopathologically confirmed to have invasive breast cancer without axillary LN metastasis. The cohort was randomly split into training and validation groups in a 7:3 ratio. Radiomics features for each lesion were extracted from the first post-contrast dynamic contrast-enhanced (DCE)-MRI. The Least Absolute Shrinkage and Selection Operator (LASSO) regression method and logistic regression analyses were employed to identify significant radiomic features and clinicoradiological variables. These models were established using four machine learning (ML) algorithms and one DL algorithm. The predictive performance of the models (radiomics, clinicoradiological, and combination) was assessed through discrimination and compared using the DeLong test. Four clinicoradiological parameters and 10 radiomic features were selected by LASSO for model development. The Multilayer Perceptron (MLP) model, constructed using both radiomic and clinicoradiological features, demonstrated excellent performance in predicting LVI, achieving a high area under the curve (AUC) of 0.835 for validation. The DL model (MLP-radiomic) achieved the highest accuracy (AUC = 0.896), followed by DL model (MLP-combination) with an AUC of 0.835. Both DL models were significantly superior to the ML model (RF-clinical) with an AUC of 0.720. The DL model (MLP), which integrates radiomic features with clinicoradiological information, effectively aids in the preoperative determination of LVI status in patients with invasive breast cancer and negative axillary LNs. This is beneficial for making informed clinical decisions.

## Introduction

Lymphovascular invasion (LVI), defined as the infiltration of cancer cells into the lymphatic or vascular intraluminal area at the periphery of the primary tumor site^1^, is widely recognized as an unfavorable prognostic factor for patients with lymph node (LN)-negative breast cancer^2^. LVI is a crucial step in LN metastasis, and LVI-positive tumors have a higher potential for local recurrence and distant metastasis than LVI-negative ones^3–5^. Specifically, for patients with LN-negative breast cancer, preoperative detection of LVI status can predict metastatic potential, informing decisions regarding adjuvant treatment^6–8^. Comprehensive regional nodal irradiation is recommended for patients with LVI according to the NCCN guidelines^9^. Furthermore, the presence of LVI is related to non-sentinel lymph node involvement in patients with a positive sentinel node biopsy^10^. Currently, LVI status is determined using surgical pathology specimens, which can lead to complications such as delayed treatment, additional surgeries, unnecessary anxiety, and diminished quality of life. Therefore, early identification of LVI during treatment is crucial. While preoperative needle biopsy is the preferred method for most patients to determine tumor characteristics, accurate detection of LVI through partial sampling from core biopsy is challenging due to tissue shrinkage and mechanically induced cellular displacement^11^.

Multiparametric MRI is an exceptionally reliable, comprehensive, and accurate diagnostic technique for preoperative staging, boasting the highest sensitivity and a reasonably high specificity for detecting breast malignancy among current imaging patterns^12,13^. Several studies on breast MRI have indicated that certain MRI features such as mass margins, adjacent-vessel sign (AVS), peritumoral edema, tumor size, background parenchymal enhancement (BPE), apparent diffusion coefficient (ADC) patterns, and contrast enhancement patterns are associated with LVI^2,12,14,15^. Nevertheless, their results are sometimes inconsistent due to subjective factors during feature assessment.

Radiomics, a recent branch of science, can aid radiologists in making highly accurate diagnoses through deep mining, prediction, and analysis of large data sets^16^. Deep learning (DL), in contrast, has made significant strides in the field of breast imaging diagnosis, particularly in the detection and classification of breast cancer^17^.

While several studies have utilized MRI-based radiomics to predict LVI in patients with breast cancer^18–21^, the cohort sizes in these studies were small, potentially affecting the outcomes. To the best of our knowledge, few studies have integrated the clinicoradiological features with DL signatures to establish LVI-predictive models^19,21,22^, and data on DL techniques for determining LVI status in a node-negative invasive breast cancer cohort are scarce.

Accordingly, in this study, we collected data from a large cohort (280 patients). Our aim was to investigate whether a DL model incorporating MRI-based radiomic features, clinicopathologic characteristics, and conventional MRI manifestations could serve as a preoperative tool for LVI prediction in patients with invasive breast cancer and negative axillary LNs.

## Materials and methods

### Patients

**Figure 1:**
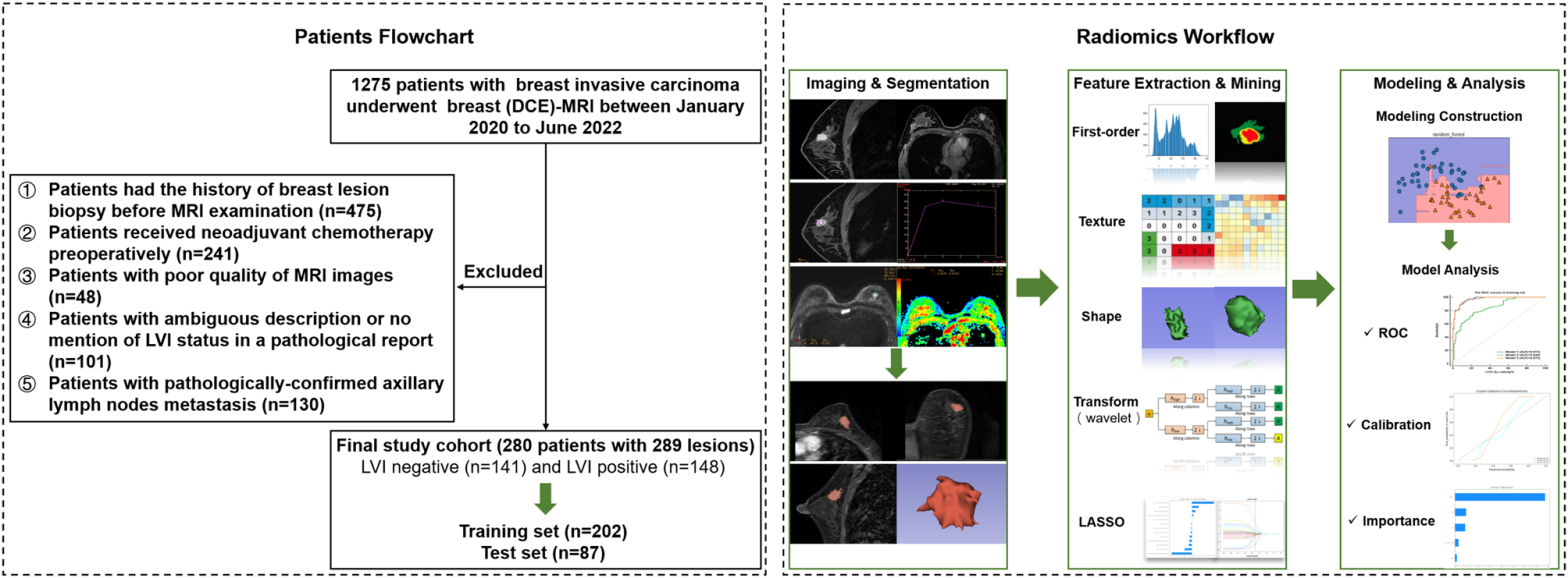
The overall workflow of the study. The patient recruitment and random grouping is displayed in the left dotted box. The radiomics workflow is displayed in the right dotted box.

This retrospective study was approved by the Tianjin cancer hospital medical ethics committee, which waived the requirement for informed consent and all research was performed in accordance with relevant guidelines. We recruited a total of 1275 consecutive female patients diagnosed with invasive breast carcinoma between January 2020 and June 2022. The exclusion criteria are detailed in Appendix E1. Ultimately, 280 patients with 289 lesions were included. These patients were randomly grouped into training (n = 202) and validation (n = 87) datasets at a ratio of 7:3. The overall study design is illustrated in Fig. 1.

### Image acquisition protocol

MRI examinations were conducted with patients in the prone position using a 1.5 T scanner (Signa HDxt, GE Healthcare, USA) and a 3.0 T scanner (Signa Pioneer and Discovery 750, GE Healthcare, USA), each equipped with a dedicated four- or eight-channel phased-array breast coil. Our center’s routine protocols included the following sequences: axial fast spin-echo (FSE) T1-weighted imaging (T1WI), axial fat-suppressed spin-echo T2-weighted imaging (T2WI), axial echo-planar diffusion-weighted imaging (DWI), and a sagittal dynamic contrast-enhanced (DCE) sequence. Six dynamic sagittal sequences were acquired once before and five times after the injection of contrast medium (Gd-DTPA, 0.2 mL/kg body weight, flow rate 2.0 mL/s) at 60-s intervals, immediately followed by an axial delayed-enhancement sequence. The relevant parameters for each sequence are detailed in Appendix E2.

### Image interpretation

Two radiologists, each with 6 and 8 years of experience in breast imaging diagnosis, independently analyzed all MRI manifestations according to the 2013 Breast Imaging Reporting and Data System (BI-RADS) lexicon of the American College of Radiology. Both observers were blinded to the histopathological information. They evaluated conventional imaging findings of the lesions, including lesion type, tumor size, lesion location, tumor margin, time-intensity curve (TIC) patterns, BPE, AVS, and tumor ADC value. All images were sent to the Advantage Workstation (AW 4.6 and AW4.7, GE Healthcare) for further post-processing, including TIC and ADC value measurements, using Functool and READY View software. Circular ROIs, accounting for approximately 75% of each lesion after avoiding areas with hemorrhage or necrosis, were drawn and used. TIC patterns were assessed and categorized into three types (I, II, and III): progressive enhancement, plateau, and washout patterns. BPE was divided into two categories: extremely minimal (or mild enhancement) and moderate (or marked) enhancement. Peritumoral edema was defined as high signal intensity surrounding the tumor on T2WI^23^. AVS positivity was defined as either one or more vessels entering the lesion on the T1WI post-enhancement sequence^24^. The largest tumor diameter in the image showing the largest lesion was measured and recorded. For the semi-quantitative kinetic curve parameters automatically computed by the Functool software, we measured SI_0_ (signal intensity values in the pre-contrast images), SI_1_ (signal intensity values in the first post-contrast images), and SI_max_ (the maximum signal intensity values in the post-contrast images). The early enhancement ratio (EER) was calculated as follows: EER = (SI_1_ − SI_0_)/ SI_0_ × 100. The peak enhancement ratio (PER) was calculated as follows: PER = (SI_max_ − SI_0_)/SI_0_ × 100. Time-to-peak enhancement (TTP) was also obtained. The reliability of the observations was assessed using the intraclass correlation coefficient (ICC). Features with ICCs larger than 0.75 were considered to have satisfactory reproducibility and were reserved for further analysis.

### Histopathological assessment

The expression levels of estrogen receptor (ER), progesterone receptor (PR), human epidermal growth factor receptor 2 (HER2), and Ki-67, along with the presence of IMPCs, were pathologically evaluated using hematoxylin and eosin (H&E) and immunohistochemical analysis. ER and PR were considered positive if their expression level was greater than 1%. HER2 was considered negative if its value was 0 or 1, and positive if its value was 3 or greater. If the HER2 value was 2, fluorescence in situ hybridization (FISH) was required for further confirmation of HER2 status. High expression of Ki-67 was defined as ≥ 14%, and low expression as < 14%. All cases were classified into four immunohistochemical subtypes based on the 2013 St. Gallen Consensus Conference^25^: luminal A, luminal B, HER2-positive, and triple-negative.

### Volume of interest (VOI) segmentation and radiomic feature extraction

Segmentations for all MRI images were performed by a radiologist with 6 years of experience in MRI interpretation. Subsequently, 100 randomly selected cases were assigned to another radiologist with 8 years of experience in MRI interpretation. Both radiologists were blinded to the patients’ clinical data. An example of the segmentation process is shown in Fig. S1. Feature extraction was performed using the radiomics module in the 3D Slicer software. A total of 851 radiomic features were extracted from each ROI. These features included 107 original features and 744 wavelet-based features (comprising first-order statistics, gray-level co-occurrence matrix [GLCM], gray-level size-zone matrix [GLSZM], gray-level run-length matrix [GLRLM], gray-level dependence matrix [GLDM], neighboring gray tone difference matrix [NGTDM], and shape-based features). A description of the initial 851 features is provided in Appendix E3.

### Radiomic and clinicoradiological feature selection

Feature selection and ML/DL model development were performed on the training cohort, while the validation cohort was kept completely independent and invisible until the final model performance was evaluated. Feature standardization (standard deviation) was performed before feature selection. The selection of robust radiomic features included three steps. First, to reduce the risk of overfitting, features with highly pairwise correlations at the level of |r| ≥ 0.9 were removed by Pearson’s correlation analysis. Second, we performed univariate selection using the SelectKBest method for every feature, and features with p values < 0.05 were retained for further analysis. Third, the Least Absolute Shrinkage and Selection Operator (LASSO) method was adopted to identify the optimal log (λ) value by fivefold cross-validation and obtain the robust radiomic features with a non-zero coefficient for the differentiation of the LVI and non-LVI groups. Independent clinicoradiological features were selected from the variables found to be significant in the univariate analysis using multivariate logistic regression analysis.

### Model construction and validation

Four different ML classification algorithms (random forest (RF), logistic regression (LR), support vector machine (SVM) and stochastic gradient descent (SGD)), and a DL classification algorithm (Multilayer Perceptron (MLP)) were used to build models for predicting LVI in the training cohort. We tuned the parameters of the different algorithms using fivefold cross-validation. The optimal hyperparameters for each algorithm were obtained based on the evaluation results. The relevant hyperparameters are presented in Appendix E4. Predictive models for the radiomic signature (Model 1), selected clinicoradiological variables (Model 2), and integrated features (combining the above two parameters, Model 3) were constructed for the training cohort and were validated in the validation cohorts.

The models’ performance was evaluated based on discrimination metrics, which included the receiver operating characteristic (ROC) curve, area under the ROC curve (AUC), accuracy, sensitivity, specificity, positive predictive value (PPV), and negative predictive value (NPV). The AUCs of the models were compared using DeLong’s test.

### Statistical analysis

Statistical analyses were conducted using MedCalc^®^ version 20.0.3 (MedCalc Software Ltd, Ostend, Belgium) and R software version 4.2.2 (http://www.Rproject.org). Continuous variables are presented as the mean ± standard deviation (SD), and categorical variables as frequencies or percentages, unless otherwise specified. The Kolmogorov–Smirnov test was utilized to test the normality of the data distribution. Continuous variables were compared using the Mann–Whitney *U* test, while dichotomous qualitative variables were assessed using the Chi-squared test or Fisher’s exact test. Univariate and multivariate logistic regression methods were employed to determine the association between clinicopathological and radiological features and LVI. Radiomic feature selection and model development were performed using the Shukun Medical Research Platform (https://medresearch.shukun.net/project; Appendix E5). All tests were performed two-sided, and a p-value of < 0.05 was considered statistically significant.

## Results

### Patient and MRI characteristics

**Figure 2:**
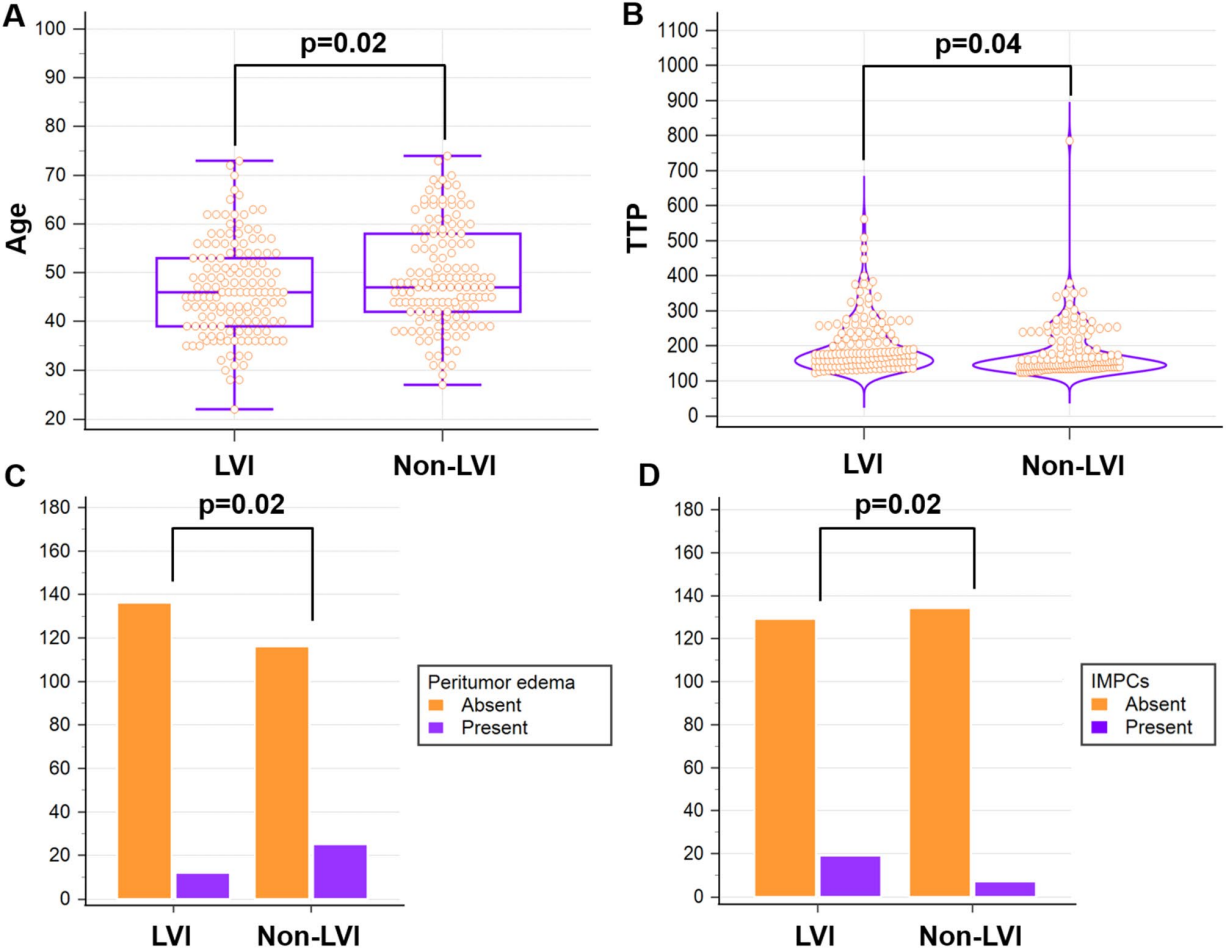
Significant clinicoradiological features. (**A**) patient age, (**B**) TTP, (**C**) peritumor edema, and (**D**) IMPCs selected by logistic regression analysis.

The ICCs for all MRI characteristics exceeded 0.75 between the two radiologists. A comparison of the clinicoradiological features between the LVI-positive and LVI-negative patient groups is presented in Table 1. In total, 280 consecutive patients were included (median age, 47 years; range, 22–74 years). LVI was found in 140 patients with 148 lesions and was absent in 140 patients with 141 lesions. In the univariate analysis, patient age, peritumoral edema, TTP, and IMPCs were significantly correlated with LVI (p < 0.05). In the multivariate logistic regression analysis, patient age (odds ratio [OR] = 1.03; 95% confidence interval [CI] 1.00–1.05; p = 0.02), peritumoral edema (OR = 2.44; 95% CI 1.18–5.08; p = 0.02), TTP (OR = 1.00; 95% CI 0.99–1.00; p = 0.04) and IMPCs (OR = 0.35; 95% CI 0.14–0.87; p = 0.02) remained independent predictors of LVI (Table 2 and Fig. 2).

**Table 1:** Clinicopathological and MRI characteristics of breast cancer patients with or without LVI.

Characteristic	Overall	LVI (n = 148)	Non-LVI (n = 141)	P value
Age, y	47.00 (40.00–55.00)	46.00 (39.00–53.00)	47.00 (42.00–58.00)	0.034
Menopausal status				0.098
Premenopausal	186 (64.4%)	102 (68.9%)	84 (59.6%)	
Postmenopausal	103 (35.6%)	46 (31.1%)	57 (40.4%)	
Maximum diameter, cm	2.20 (1.70–3.10)	2.20 (1.70–3.20)	2.30 (1.70–2.80)	0.920
Lesion type				0.417
Mass	230 (79.6%)	115 (77.7%)	115 (81.6%)	
NME	59 (20.4%)	33 (22.3%)	26 (18.4%)	
TIC pattern				0.499
Type I	2 (0.7%)	2 (1.4%)	0 (0.0%)	
Type III and Type II	287 (99.3%)	146 (98.6%)	141 (100.0%)	
ADC value (mm^2^/s)	0.86 (0.79–0.97)	0.87 (0.79–0.95)	0.86 (0.78–0.99)	0.993
Peritumoral edema				0.041
Present	55 (19.0%)	35 (23.6%)	20 (14.2%)	
Absent	234 (81.0%)	113 (76.4%)	121 (85.8%)	
AVS				0.618
Present	233 (80.6%)	121 (81.8%)	112 (79.4%)	
Absent	56 (19.4%)	37 (18.2%)	29 (20.6%)	
Intratumoral T2 high signal				0.078
Present	48 (16.6%)	19 (12.8%)	29 (20.6%)	
Absent	241 (83.4%)	129 (87.2%)	112 (79.4%)	
EER (%)	209.00 (178.75–237.00)	211.00 (177.00–237.00)	205.00 (179.75–236.25)	0.956
PER (%)	213.00 (182.00–239.00)	214.00 (181.00–238.50)	212.00 (184.75–242.00)	0.952
TTP (s)	162.00 (142.00–230.00)	175.00 (148.00–236.00)	154.00 (139.00–214.00)	0.002
BPE				0.076
Minimal or mild	185 (64.0%)	102 (68.9%)	83 (58.9%)	
Moderate or marked	104 (36.0%)	46 (31.1%)	58 (41.1%)	
DWI rim sign				0.188
Present	159 (55.0%)	87 (58.8%)	69 (48.9%)	
Absent	130 (45.0%)	61 (41.2%)	72 (51.1%)	
Histology				0.563
IDC	255 (88.2%)	129 (87.2%)	126 (89.4%)	
Mixed or other	34 (11.8%)	19 (12.8%)	15 (10.6%)	
ER status				0.053
Negative	36 (12.5%)	13 (8.8%)	23 (16.3%)	
Positive	253 (87.5%)	135 (91.2%)	118 (83.7%)	
PR status				0.479
Negative	73 (25.3%)	40 (27.0%)	33 (23.4%)	
Positive	216 (74.7%)	108 (73.0%)	108 (76.6%)	
HER2 status				0.982
Negative	242 (83.7%)	124 (83.8%)	118 (83.7%)	
Positive	47 (16.3%)	24 (16.2%)	23 (16.3%)	
Ki-67 status				0.181
High (≥ 14%)	194 (67.1%)	94 (63.5%)	100 (70.9%)	
Low (< 14%)	95 (32.9%)	54 (36.5%)	41 (29.1%)	
AR status				0.351
Negative	47 (16.3%)	27 (18.2%)	20 (14.2%)	
Positive	242 (83.7%)	121 (81.8%)	121 (85.8%)	
Histological grade				0.850
I	14 (4.8%)	4 (2.7%)	10 (7.1%)	
II	204 (70.6%)	110 (74.3%)	94 (66.7%)	
III	71 (24.6%)	34 (23.0%)	37 (26.2%)	
Molecular subtype				0.153
Luminal A	68 (23.5%)	35 (23.6%)	33 (23.4%)	
Luminal B	185 (64.0%)	100 (67.6%)	85 (60.3%)	
HER2 positive	13 (4.5%)	3 (2.0%)	10 (7.1%)	
Triple negative	23 (8.0%)	10 (6.8%)	13 (9.2%)	
IMPCs				0.020
Present	26 (9.0%)	19 (12.8%)	7 (5.0%)	
Absent	263 (91.0%)	129 (87.2%)	134 (95.0%)	

*LVI* lymphovascular invasion, *NME* non-mass enhancement, *TIC* time-intensity curve, *ADC* apparent diffusion coefficient, *AVS* adjacent vessel sign, *EER* early enhancement ratio, *PER* peak enhancement ratio, *TTP* time to peak, *BPE* background parenchymal enhancement, *IDC* invasive ductal carcinoma, *ER* estrogen receptor, *PR* progesterone receptor, *HER-2* human epidermal growth factor receptor 2, *AR* androgen receptor, *IMPCs* invasive micropapillary components.

**Table 2:** Univariable and multivariable logistic regression analyses for clinicopathological and MRI characteristics in primary cohort (n = 289).

Variables	Univariable analysis		Multivariable analysis	
	OR (95% CI)	P value	OR (95% CI)	P value
Age, y	0.974 (0.951, 0.997)	0.024	0.958 (0.934, 0.983)	0.001
Peritumoral edema	2.117 (1.063, 4.213)	0.033	2.474 (1.191, 5.142)	0.015
TTP (s)	1.004 (1.000, 1.007)	0.037	1.004 (1.001, 1.007)	0.024
IMPCs	2.820 (1.147, 6.933)	0.024	3.205 (1.235, 8.317)	0.017

*TTP* time to peak, *IMPCs* invasive micropapillary components.

### Radiomic feature selection

**Figure 3:**
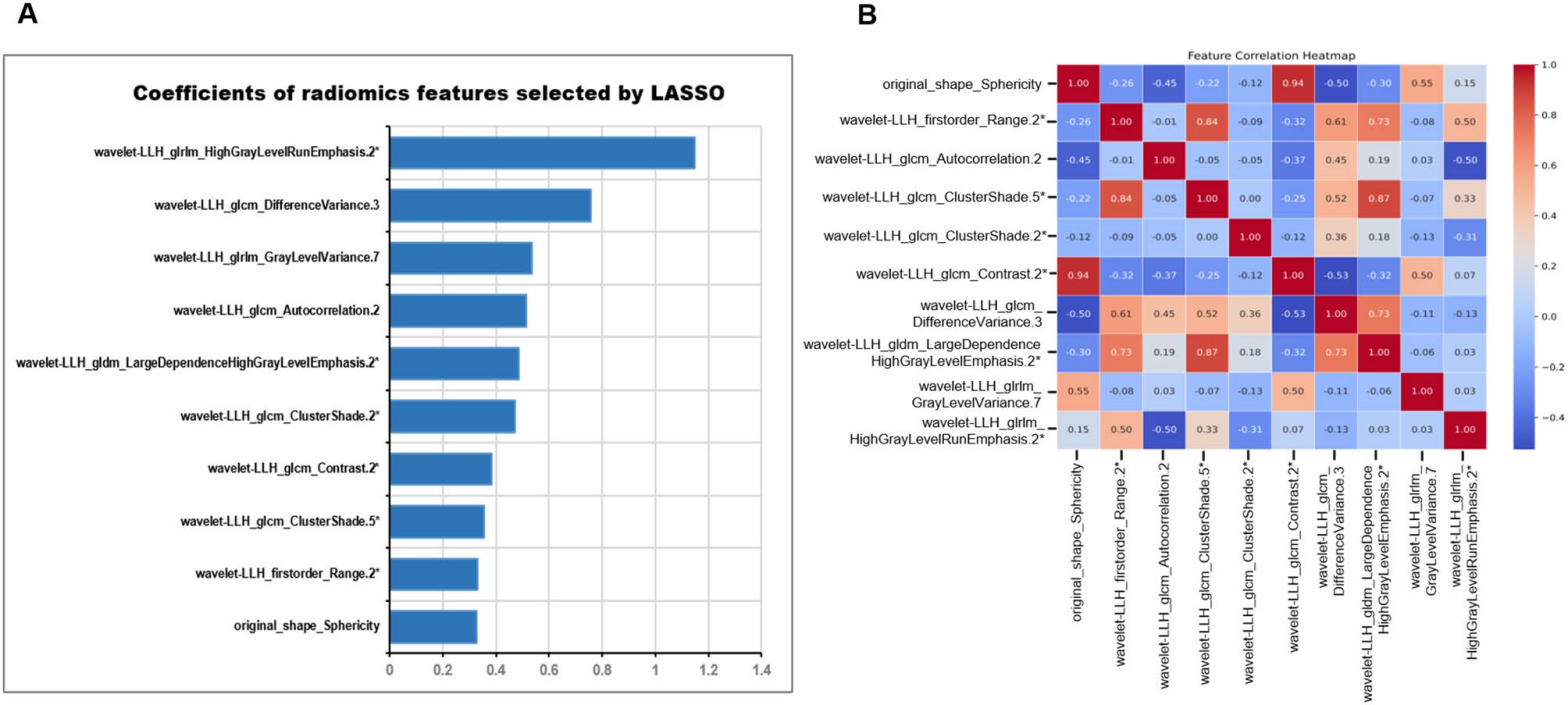
Feature-weighted diagram of LASSO model with 10 features included in model (**A**) and feature correlation plot (**B**). *The LASSO coefficient is a negative value, and the radiomic features are arranged in descending order according to the absolute value of the LASSO coefficient.

In this study, a total of 851 radiomics features for each lesion were extracted, including 107 original features and 744 wavelet-based features. 282 features remained after feature preselection using the Pearson correlation analysis. Then, using the single-variable feature selection method (SelectKBest), 117 features remained. After further feature dimensionality reduction by the LASSO regression algorithm, 10 significant radiomic features were finally selected for the subsequent establishment of the ML and DL models (Figs. S2 and Fig. 3).

### Predictive models assessment

**Figure 4:**
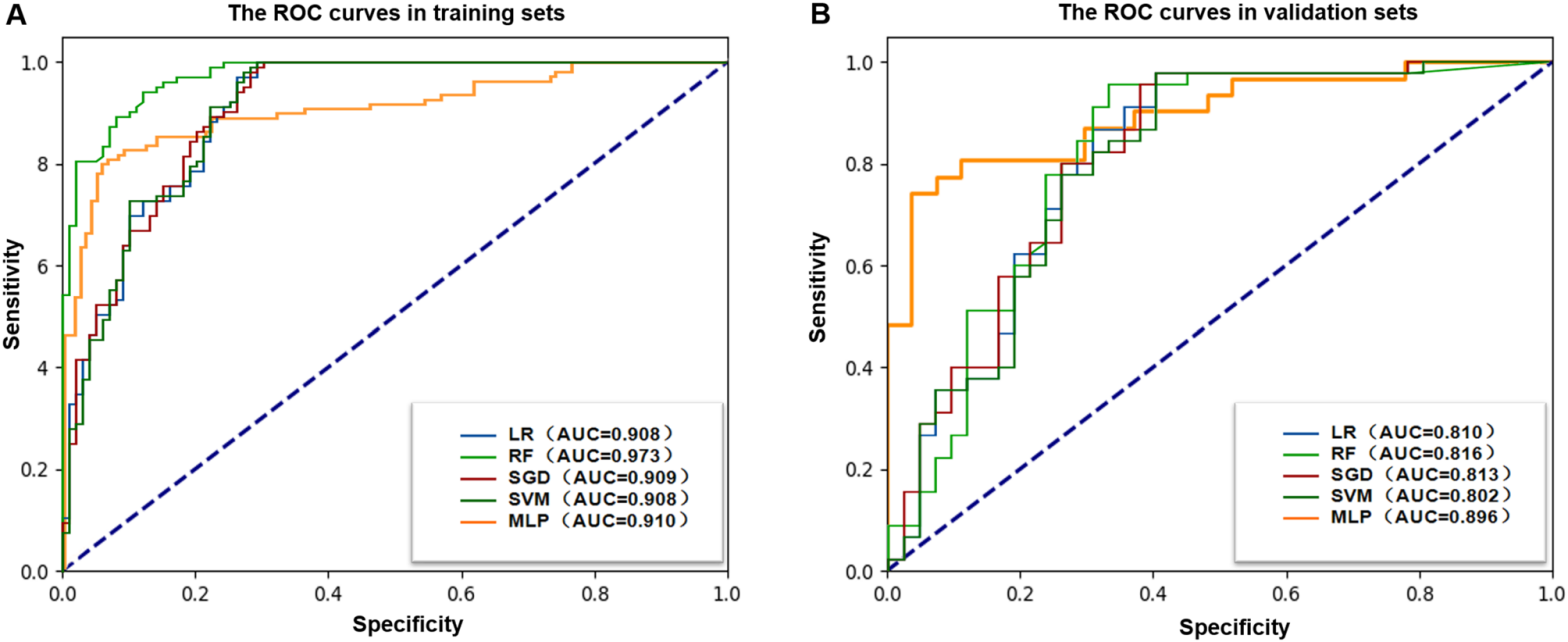
The ROC curves of radiomics models based on five classifiers in (**A**) the training cohorts and (**B**) the validation cohorts.

Radiomics predictive models were built using different ML algorithms and a DL algorithm, which demonstrated good diagnostic performance in the training sets. The predictive performance was validated using the validation dataset. The optimal model was the MLP model (AUC = 0.896; 95% CI 0.806–0.964). The corresponding ROC curves, AUC, accuracy, sensitivity, specificity, PPV, and NPV values are shown in Table 3 and Fig. 4.

**Table 3:** Diagnostic performance of radiomics models based on four different ML algorithms and MLP algorithm.

Models	AUC	95% CI		Sensitivity	Specificity	Accuracy	PPV	NPV
		Lower	Upper					
LR								
Training set	0.908	0.868	0.949	0.737	0.971	0.856	0.961	0.794
Validation set	0.810	0.714	0.906	0.691	0.867	0.782	0.829	0.750
RF								
Training set	0.973	0.956	0.990	0.879	0.942	0.911	0.936	0.890
Validation set	0.816	0.720	0.912	0.714	0.800	0.759	0.769	0.750
SGD								
Training set	0.909	0.869	0.949	0.717	0.981	0.852	0.973	0.783
Validation set	0.813	0.719	0.906	0.619	0.867	0.747	0.813	0.709
SVM								
Training set	0.908	0.867	0.949	0.727	0.981	0.856	0.973	0.789
Validation set	0.802	0.705	0.899	0.595	0.889	0.747	0.833	0.702
MLP*								
Training set	0.910	0.871	0.948	0.891	0.702	0.792	0.731	0.876
Validation set	0.896	0.806	0.964	0.806	0.814	0.810	0.833	0.786

*AUC* area under the ROC curves, *CI* confidence interval, *PPV* positive prediction value, *NPV* negative prediction value, *LR* logistic regression, *RF* random forest, *SGD* stochastic gradient descent, *SVM* support vector machine, *MLP* multilayer perceptron.

*The optimal predictive performance in the validation set was observed in the MLP model.

The models based on the selected clinicoradiological variables applied to the four ML algorithms and MLP algorithm showed relatively good performance in predicting LVI after fivefold cross-validation. The best performance was observed in the RF model (AUC = 0.720; 95% CI 0.611–0.830). The corresponding ROC curves, AUC, accuracy, sensitivity, specificity, PPV, and NPV values are presented in Table S1 and Fig. S3.

**Figure 5:**
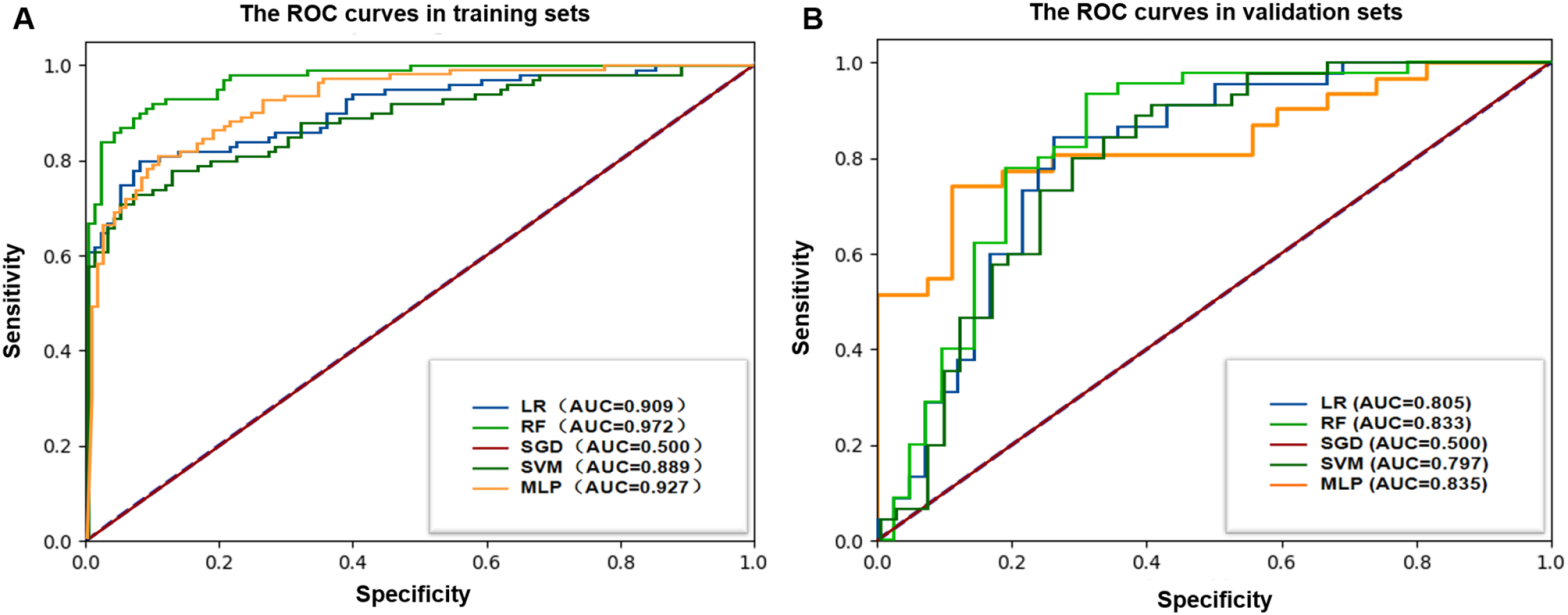
The ROC curves of combined models based on five classifiers in (**A**) the training cohorts and (**B**) the validation cohorts.

In the models based on combined features, including 10 selected radiomic signatures and f4 clinicoradiological variables, the MLP model (AUC = 0.835; 95% CI 0.719–0.929) was the optimal model after fivefold cross-validation. The corresponding ROC curves, AUC, accuracy, sensitivity, specificity, PPV, and NPV values are shown in Table 4 and Fig. 5.

**Table 4:** Diagnostic performance of combined models based on radiomic and clinicoradiological variables applied to 4 different ML algorithms and MLP.

Models	AUC	95% CI		Sensitivity	Specificity	Accuracy	PPV	NPV
		Lower	Upper					
LR								
Training set	0.909	0.869	0.950	0.798	0.922	0.861	0.908	0.826
Validation set	0.805	0.708	0.901	0.7381	0.800	0.770	0.775	0.766
RF								
Training set	0.972	0.954	0.990	0.919	0.903	0.911	0.901	0.921
Validation set	0.833	0.742	0.925	0.810	0.733	0.770	0.739	0.805
SGD								
Training set	0.500	0.500	0.500	1.000	0.000	0.490	0.490	0.000
Validation set	0.500	0.500	0.500	1.000	0.000	0.483	0.483	0.000
SVM								
Training set	0.889	0.843	0.934	0.727	0.932	0.832	0.911	0.781
Validation set	0.797	0.699	0.895	0.667	0.822	0.747	0.778	0.726
MLP*								
Training set	0.927	0.893	0.955	0.991	0.380	0.671	0.592	0.979
Validation set	0.835	0.719	0.929	0.935	0.333	0.655	0.617	0.818

*AUC* area under the ROC curves, *CI* confidence interval, *PPV* positive prediction value, *NPV* negative prediction value, *LR* logistic regression, *RF* random forest, *SGD* stochastic gradient descent, *SVM* support vector machine, *MLP* multilayer perceptron.

*The optimal predictive performance in the validation set was observed in the MLP model.

**Figure 6:**
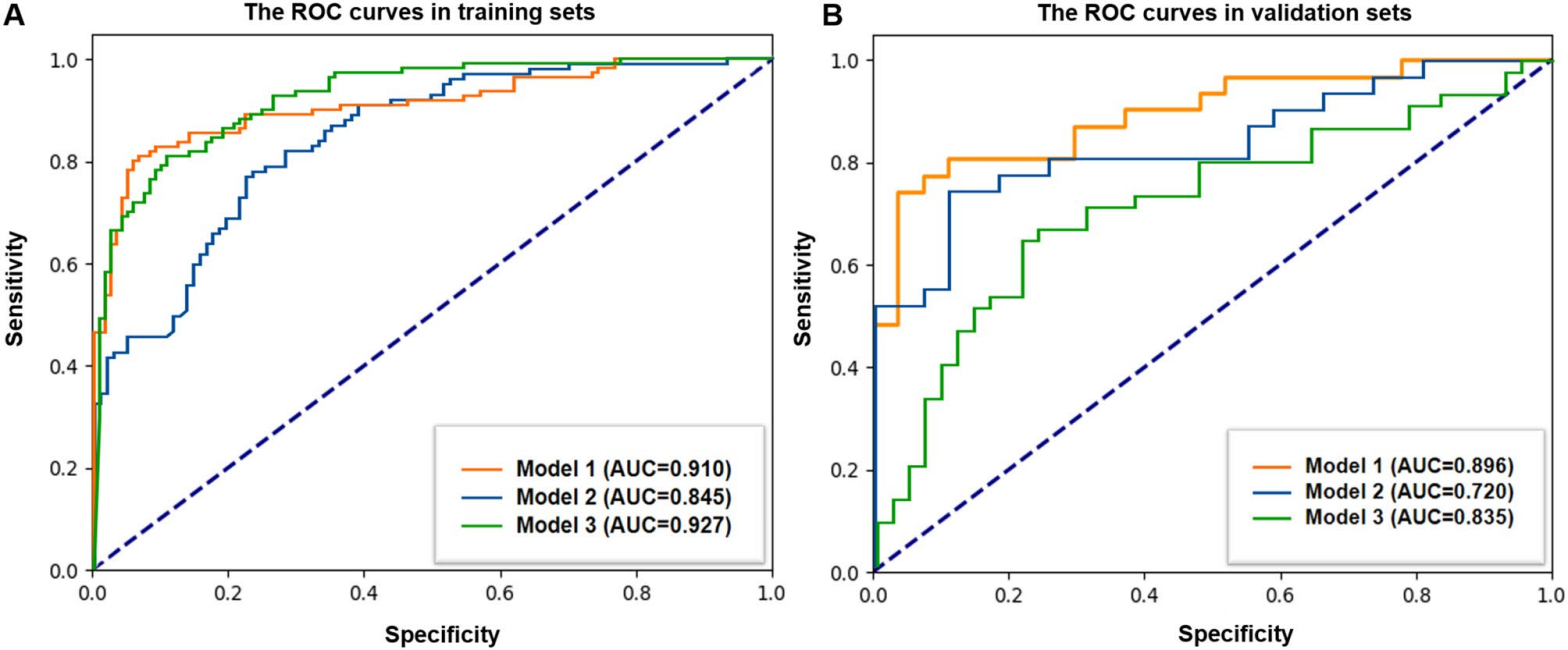
The ROC curves of the optimal models based on radiomic signatures alone (Model 1), clinicoradiological variables (Model 2) and combined features including the above two (Model 3) in (**A**) the training cohorts and (**B**) the validation cohorts.

The performances of model 1, 2 and 3 in the optimal classifier (RF and MLP algorithm) are displayed in Fig. 6. In the validation cohort, the Delong test showed that the model 1 achieved the highest accuracy (AUC = 0.896), being significantly superior to the model 2 (AUC = 0.720) or model 3 (AUC = 0.835) (p < 0.001). Model 3 exhibited better performance than models 2 (p < 0.001) (Table 5).

**Table 5:** Comparation on the performances of model 1, model 2 and model 3 in identifying LVI in the training and validation cohorts.

Models	AUC	95% CI		Sensitivity	Specificity	Accuracy	P value
		Lower	Upper				
Model 1							< 0.001*
Training set	0.910	0.871	0.948	0.891	0.702	0.792	
Validation set	0.896	0.806	0.964	0.806	0.814	0.810	
Model 2							< 0.001*
Training set	0.845	0.793	0.897	0.778	0.767	0.772	
Validation set	0.720	0.611	0.830	0.762	0.667	0.713	
Model 3							
Training set	0.927	0.893	0.955	0.991	0.380	0.671	
Validation set	0.835	0.719	0.929	0.935	0.333	0.655	

*AUC* area under the ROC curves, *CI* confidence interval.

*DeLong test for comparing with model 3 in each validation set.

## Discussion

In this study, we investigated the relationship between LVI and invasive breast cancer in node-negative patients. We constructed MRI-based radiomic models that demonstrated a highly robust ability to predict LVI. Four different ML algorithms and one DL algorithm were used for model construction based on the 10 selected radiomic signatures, and the DL model outperformed the other models in terms of identifying the LVI status. Furthermore, the combined model, which integrated clinicoradiological characteristics with radiomic signatures, exhibited significantly better predictive performance than the clinical model. This is expected to offer a relatively accurate and objective approach for preoperative LVI prediction.

Some studies have shown that LVI is associated with age, tumor size, mass margins, positive lymph nodes, BPE, TIC patterns, ADC values, peritumoral edema, AVS, DWI rim sign, and histologic grade^1,2,12,15,26^. Our study found that the risk of LVI increased with a lower mean age. The mean age of the patients with LVI was lower than that of the patients without LVI. A previous study revealed that age is a high-risk factor for LVI in invasive breast cancer^27^. Yang et al.^28^ reported that premenopausal status was associated with an increased risk (4.59-fold) of early-onset breast cancer (< 40 years). Early-onset breast cancer is more aggressive in premenopausal women than in older women, due to the gradual decrease in blood estrogen and progesterone levels with age^29^.

We also identified peritumoral edema as a predictor of LVI; patients with peritumoral edema had a higher risk of LVI, which aligns with previous study results^21,26^. A significantly higher incidence of peritumoral edema was observed in patients with more biologically aggressive tumors^23^. One possible reason for peritumoral edema may be an increase in vascular permeability and obstruction of lymphatic drainage by tumor emboli^30^. TTP, a semiquantitative DCE-MRI parameter, was found to be an independent predictive factor for LVI. We found that patients with a longer TTP had a higher probability of positive LVI, which might be related to the reduced focal blood flow caused by tumor emboli. Numerous studies have shown that TTP is a diagnostic indicator of malignant breast lesions^31^ and an independent predictive factor of pathological complete response (pCR) after neoadjuvant chemotherapy (NAC)^32–34^. One recent study analyzed the relationship between TTP and LVI, but found no significant differences between patients with and without LVI^35^. Our study identified IMPC as a predictive indicator of LVI. To the best of our knowledge, this is the first study to include IMPCs to construct a predictive model for detecting the LVI status in patients with invasive breast cancer. IMPC is a rare subtype of an epithelial tumor of the breast listed in the 2003 World Health Organization (WHO) histological classification of breast tumors^36^. Previous studies demonstrated that breast cancers with IMPCs have a relatively high incidence of LVI^37^. LVI has also been found to be more common among patients with IMPC (pure or mixed with IDC), as reported by Tang et al.^38^, with 14.7% versus only 0.1% in the IDC group and 94.7% versus 71.9% in the control group reported by Gokce et al.^39^. In our study, we found a higher frequency of IMPC in patients with LVI than in those without LVI, which is consistent with a previous study^40^.

Several studies have confirmed that the DCE-MRI-based radiomics ML model has robust power for predicting the LVI status of patients with invasive breast cancer^18–21^. Zhang et al.^21^ identified that the proposed nomogram, incorporating MRI-based radiomic signatures and MRI-reported peritumoral edema, achieved satisfactory preoperative prediction of LVI and clinical outcomes in patients with IDC. A recent study used an SVM classifier to establish a prediction model for LVI based on the ADC radiomic signature, and its AUC was 0.77 in the test set^20^. Liu and colleagues^19^ confirmed that a radiomics model based on DCE-MRI utilizing a multivariate logistic regression method significantly improved the performance for discriminating LVI-positive from LVI-negative lesions. Another study found that an ML-based radiomics model based on 3D segmentation of ADC maps could be used to predict the LVI status in breast cancer patients^18^. These studies, however, only employed classical non-network methods for classification. In contrast, our research introduced and a deep learning model (MLP) for comparison. Yang et al.^22^ developed a diagnostic model that combined MRI morphological features, Radiomics, and DL features to determine the LVI status in 206 breast cancer patients. They found that the combined model was more efficient in distinguishing between LVI positive and negative cases compared to individual models. A recent study^41^ developed a novel DL framework, “Prior Clinico-Radiological Features Informed Multi-Modal MR Images Convolutional Neural Network (PCMM-Net)”, for predicting LVI in breast cancer, achieving a higher AUC of 0.843 than clinico-radiological features alone. This aligns with our study’s findings. However, their studies included both lymph node-positive and -negative patients, while ours only included lymph node-negative patients. We selected this sample group due to its clinical significance. The 16th St. Gallen consensus established that the inclusion of chemotherapy for N0 patients should also be based on the presence of LVI^42^. LVI is also currently of interest for predicting non-sentinel lymph node metastases^43^. In clinical practice, axillary node dissection is typically recommended for effective local treatment of patients with positive sentinel nodes^44^. Nevertheless, since over 40% of patients with positive sentinel nodes have no other nodal metastases, predicting non-sentinel node involvement has been conducive to avoiding unnecessary axillary node dissection^45^. Consequently, the prediction of non-sentinel node involvement associated with the presence of LVI might allow for appropriate treatment (sentinel node biopsy or axillary node dissection) of patients.

This study has several limitations. First, this was a retrospective single-center study, highlighting the necessity for a large-scale prospective multicenter study to further validate the effectiveness of the proposed radiomics model. Second, only the first phase after enhancement was used to extract radiomic features in this study, suggesting the need for other sequences, such as T2W, DWI images, and ADC maps, for feature extraction in further studies. Third, our institution originally designed a sagittal DCE sequence for breast MRI examination, meaning the functional tumor volume (FTV) of the lesions could not be obtained. Similarly, our study only analyzed semi-quantitative kinetic curve parameters for LVI prediction. Quantitative pharmacokinetic parameters (such as ktrance, kep, and Ve) were not measured using our equipment. Further exploration of these parameters is required, if conditions permit.

In conclusion, the radiomic features derived from DCE-MRI are robust biomarkers for predicting LVI. The combined DL model, incorporating the radiomic signature and clinicoradiological-based variables, exhibited a highly acceptable predictive efficacy for LVI status in patients with node-negative invasive breast cancer.

### Supplementary Information

Supplementary Information.

## Data Availability

The datasets used and/or analysed during the current study available from the corresponding author on reasonable request.

## Abbreviations

ADC: Apparent diffusion coefficient
AR: Androgen receptor
AUC: Area under the curve
AVS: Adjacent vessel sign
BI-RADS^®^: Breast Imaging Reporting and Data System
BPE: Background parenchymal enhancement
DL: Deep learning
EER: Early enhancement ratio
ER: Estrogen receptor
HER2: Human epidermal growth factor receptor 2
ICC: Interclass correlation coefficient
IDC: Invasive ductal carcinoma
IMPCs: Invasive micropapillary components
LASSO: Least Absolute Shrinkage and Selection Operator
LVI: Lymphovascular invasion
ML: Machine learning
NME: Nonmass enhancement
PER: Peak enhancement ratio
PR: Progesterone receptor
ROC: Receiver operating characteristic
RF: Random Forest
SLNB: Sentinel lymph node biopsy
TIC: Time-intensity curve
TTP: Time to peak

## Code availability

The essential analysis code used to produce the results is available from the GitHub repository at https://github.com/Sarah147369/BreastCancerLVI-Prediction.
