# Supplementary material for: Predictive value of MRI-based deep learning model for lymphovascular invasion status in node-negative invasive breast cancer
Source: Sci Rep. 2024 Jul 13;14:16204. doi: 10.1038/s41598-024-67217-0 (PMC11246470; doi:10.1038/s41598-024-67217-0)
Supplement: Supplementary file 1 — Supplementary Information. [file 41598_2024_67217_MOESM1_ESM.docx]

**Appendix E1 The exclusion criteria for the patient cohort**

The following exclusion criteria were conducted: patients had the history of breast lesion biopsy before MRI examination, patients received neoadjuvant chemotherapy preoperatively, patients with poor quality of MRI images due to artifacts, patients with ambiguous description or no mention of LVI status in a pathological report and patients with pathologically confirmed axillary LNs metastasis.

**Appendix E2 The relevant parameters for each imaging acquisition sequence**

The routine protocols of our center consisted of the following sequences and parameters: axial fast spin-echo (FSE) T1-weighted imaging (T1WI) (TR=622ms, TE=10ms, field of view=30 cm×30 cm, matrix=384×224, NEX=2, slice thickness=5.0mm, slice gap=0.5mm) , axial fat-suppressed spin-echo T2-weighted imaging (T2WI) ( TR=6330ms, TE=68ms, field of view=30cm×30cm, matrix=384×224, NEX=2, slice thickness=5.0mm, slice gap=0.5mm), axial echo-planar diffusion-weighted imaging (DWI) ( single shot Echo Planar Imaging, SS-EPI and multiplexed sensitivity-encoding, MUSE technique, TR=3235ms, TE=64ms, field of view=30cm×30cm, matrix=128×128, NEX=2, slice thickness=4.5mm, and slice gap=0.5mm, b values=0,500 and 1000 s/mm^2^), a sagittal DCE sequence(Parallel acquisition of three-dimensional rapid gradient echo sequence volume imaging for breast assessment technique, TR=6.1ms, TE=2.9ms, field of view=26cm×26cm, matrix=256×128, flip angle=15°, NEX=1, slice thickness=1.8mm, and slice gap=0.5mm), and an axial delayed-enhancement sequence (TR=6.1ms, TE=2.9ms, matrix=256×128, flip angle=15°, slice thickness=1.8mm, NEX=1).

**Appendix E3 Initial 851 radiomic features**

| Original features  (n=107) | First-order statistics(n=18) | 10Percentile;90Percentile;Energy;Entropy;InterquartileRange;Kurtosis;Maximum;MeanAbsoluteDeviation;Mean;Median;Minimum;Range;RobustMeanAbsoluteDeviation;RootMeanSquared;Skewness;TotalEnergy;Uniformity;Variance |
| --- | --- | --- |
|  | GLCM(n=24) | Autocorrelation;ClusterProminence;ClusterShade;ClusterTendency;Contrast;Correlation;DifferenceAverage;DifferenceEntropy;DifferenceVariance;Id;Idm;Idmn;Idn;Imc1;Imc2;InverseVariance;JointAverage;JointEnergy;JointEntropy;MCC;MaximumProbability;SumAverage;SumEntropy;SumSquares |
|  | GLSZM(n=16) | GrayLevelNonUniformity.2;GrayLevelNonUniformityNormalized.1;GrayLevelVariance.2;HighGrayLevelZoneEmphasis;LargeAreaEmphasis;LargeAreaHighGrayLevelEmphasis;LargeAreaLowGrayLevelEmphasis;LowGrayLevelZoneEmphasis;SizeZoneNonUniformity;SizeZoneNonUniformityNormalized;SmallAreaEmphasis;SmallAreaHighGrayLevelEmphasis;SmallAreaLowGrayLevelEmphasis;ZoneEntropy;ZonePercentage;ZoneVariance |
|  | GLRLM(n=16) | GrayLevelNonUniformity.1;GrayLevelNonUniformityNormalized;GrayLevelVariance.1;HighGrayLevelRunEmphasis;LongRunEmphasis;LongRunHighGrayLevelEmphasis;LongRunLowGrayLevelEmphasis;LowGrayLevelRunEmphasis;RunEntropy;RunLengthNonUniformity;RunLengthNonUniformityNormalized;RunPercentage;RunVariance;ShortRunEmphasis;ShortRunHighGrayLevelEmphasis;ShortRunLowGrayLevelEmphasis |
|  | GLDM(n=14) | DependenceEntropy;DependenceNonUniformity;DependenceNonUniformityNormalized;DependenceVariance;GrayLevelNonUniformity;GrayLevelVariance;HighGrayLevelEmphasis;LargeDependenceEmphasis;LargeDependenceHighGrayLevelEmphasis;LargeDependenceLowGrayLevelEmphasis;LowGrayLevelEmphasis;SmallDependenceEmphasis;SmallDependenceHighGrayLevelEmphasis;SmallDependenceLowGrayLevelEmphasis |
|  | NGTDM(n=5) | Busyness;Coarseness;Complexity;Contrast.1;Strength |
|  | Shape-based features(n=14) | Elongation;Flatness;LeastAxisLength;MajorAxisLength;Maximum2DDiameterColumn;Maximum2DDiameterRow;Maximum2DDiameterSlice;Maximum3DDiameter;MeshVolume;MinorAxisLength;Sphericity;SurfaceArea;SurfaceVolumeRatio;VoxelVolume |
| Wavelet-based features  (n=744) | First-order statistics(n=144) | 10Percentile.1;90Percentile.1;Energy.1;Entropy.1;InterquartileRange.1;Kurtosis.1;Maximum.1;MeanAbsoluteDeviation.1;Mean.1;Median.1;Minimum.1;firstorder_Range.1;RobustMeanAbsoluteDeviation.1;RootMeanSquared.1;Skewness.1;TotalEnergy.1;Uniformity.1;Variance.1;10Percentile.2;90Percentile.2;Energy.2;Entropy.2;InterquartileRange.2;Kurtosis.2;Maximum.2;MeanAbsoluteDeviation.2;Mean.2;Median.2;Minimum.2;Range.2;RobustMeanAbsoluteDeviation.2;RootMeanSquared.2;Skewness.2;TotalEnergy.2;Uniformity.2;Variance.2;10Percentile.3;90Percentile.3;Energy.3;Entropy.3;InterquartileRange.3;Kurtosis.3;Maximum.3;MeanAbsoluteDeviation.3;Mean.3;Median.3;Minimum.3;Range.3;RobustMeanAbsoluteDeviation.3;RootMeanSquared.3;Skewness.3;TotalEnergy.3;Uniformity.3;Variance.3;10Percentile.4;90Percentile.4;Energy.4;Entropy.4;InterquartileRange.4;Kurtosis.4;Maximum.4;MeanAbsoluteDeviation.4;Mean.4;Median.4;Minimum.4;Range.4;RobustMeanAbsoluteDeviation.4;RootMeanSquared.4;Skewness.4;TotalEnergy.4;Uniformity.4;Variance.4;10Percentile.5;90Percentile.5;Energy.5;Entropy.5;InterquartileRange.5;Kurtosis.5;Maximum.5;MeanAbsoluteDeviation.5;Mean.5;Median.5;Minimum.5;Range.5;RobustMeanAbsoluteDeviation.5;RootMeanSquared.5;Skewness.5;TotalEnergy.5;Uniformity.5;Variance.5;10Percentile.6;90Percentile.6;Energy.6;Entropy.6;InterquartileRange.6;Kurtosis.6;Maximum.6;MeanAbsoluteDeviation.6;Mean.6;Median.6;Minimum.6;Range.6;RobustMeanAbsoluteDeviation.6;RootMeanSquared.6;Skewness.6;TotalEnergy.6;Uniformity.6;Variance.6;10Percentile.7;90Percentile.7;Energy.7;Entropy.7;InterquartileRange.7;Kurtosis.7;Maximum.7;MeanAbsoluteDeviation.7;Mean.7;Median.7;Minimum.7;Range.7;RobustMeanAbsoluteDeviation.7;RootMeanSquared.7;Skewness.7;TotalEnergy.7;Uniformity.7;Variance.7;10Percentile.8;90Percentile.8;Energy.8;Entropy.8;InterquartileRange.8;Kurtosis.8;Maximum.8;MeanAbsoluteDeviation.8;Mean.8;Median.8;Minimum.8;Range.8;RobustMeanAbsoluteDeviation.8;RootMeanSquared.8;Skewness.8;TotalEnergy.8;Uniformity.8;Variance.8 |
|  | GLCM(n=206) | Autocorrelation.1;ClusterProminence.1;ClusterShade.1;ClusterTendency.1;Contrast.2;Correlation.1;DifferenceAverage.1;DifferenceEntropy.1;DifferenceVariance.1;Id.1;Idm.1;Idmn.1;Idn.1;Imc1.1;Imc2.1;InverseVariance.1;JointAverage.1;JointEnergy.1;JointEntropy.1;MCC.1;MaximumProbability.1;SumAverage.1;SumEntropy.1;SumSquares.1;DependenceEntropy.1;DependenceNonUniformity.1;DependenceNonUniformityNormalized.1;DependenceVariance.1;GrayLevelNonUniformity.3;GrayLevelVariance.3;HighGrayLevelEmphasis.1;LargeDependenceEmphasis.1;LargeDependenceHighGrayLevelEmphasis.1;LargeDependenceLowGrayLevelEmphasis.1;LowGrayLevelEmphasis.1;SmallDependenceEmphasis.1;SmallDependenceHighGrayLevelEmphasis.1;SmallDependenceLowGrayLevelEmphasis.1;Autocorrelation.2;ClusterProminence.2;ClusterShade.2;ClusterTendency.2;Contrast.4;Correlation.2;DifferenceAverage.2;DifferenceEntropy.2;DifferenceVariance.2;Id.2;Idm.2;Idmn.2;Idn.2;Imc1.2;Imc2.2;InverseVariance.2;JointAverage.2;JointEnergy.2;JointEntropy.2;MCC.2;MaximumProbability.2;SumAverage.2;SumEntropy.2;SumSquares.2;Autocorrelation.3;ClusterProminence.3;ClusterShade.3;ClusterTendency.3;Contrast.6;Correlation.3;DifferenceAverage.3;DifferenceEntropy.3;DifferenceVariance.3;Id.3;Idm.3;Idmn.3;Idn.3;Imc1.3;Imc2.3;InverseVariance.3;JointAverage.3;JointEnergy.3;JointEntropy.3;MCC.3;MaximumProbability.3;SumAverage.3;SumEntropy.3;SumSquares.3;Autocorrelation.4;ClusterProminence.4;ClusterShade.4;ClusterTendency.4;Contrast.8;Correlation.4;DifferenceAverage.4;DifferenceEntropy.4;DifferenceVariance.4;Id.4;Idm.4;Idmn.4;Idn.4;Imc1.4;Imc2.4;InverseVariance.4;JointAverage.4;JointEnergy.4;JointEntropy.4;MCC.4;MaximumProbability.4;SumAverage.4;SumEntropy.4;SumSquares.4;Autocorrelation.5;ClusterProminence.5;ClusterShade.5;ClusterTendency.5;Contrast.10;Correlation.5;DifferenceAverage.5;DifferenceEntropy.5;DifferenceVariance.5;Id.5;Idm.5;Idmn.5;Idn.5;Imc1.5;Imc2.5;InverseVariance.5;JointAverage.5;JointEnergy.5;JointEntropy.5;MCC.5;MaximumProbability.5;SumAverage.5;SumEntropy.5;SumSquares.5;Autocorrelation.6;ClusterProminence.6;ClusterShade.6;ClusterTendency.6;Contrast.12;Correlation.6;DifferenceAverage.6;DifferenceEntropy.6;DifferenceVariance.6;Id.6;Idm.6;Idmn.6;Idn.6;Imc1.6;Imc2.6;InverseVariance.6;JointAverage.6;JointEnergy.6;JointEntropy.6;MCC.6;MaximumProbability.6;SumAverage.6;SumEntropy.6;SumSquares.6;Autocorrelation.7;ClusterProminence.7;ClusterShade.7;ClusterTendency.7;Contrast.14;Correlation.7;DifferenceAverage.7;DifferenceEntropy.7;DifferenceVariance.7;Id.7;Idm.7;Idmn.7;Idn.7;Imc1.7;Imc2.7;InverseVariance.7;JointAverage.7;JointEnergy.7;JointEntropy.7;MCC.7;MaximumProbability.7;SumAverage.7;SumEntropy.7;SumSquares.7;Autocorrelation.8;ClusterProminence.8;ClusterShade.8;ClusterTendency.8;Contrast.16;Correlation.8;DifferenceAverage.8;DifferenceEntropy.8;DifferenceVariance.8;Id.8;Idm.8;Idmn.8;Idn.8;Imc1.8;Imc2.8;InverseVariance.8;JointAverage.8;JointEnergy.8;JointEntropy.8;MCC.8;MaximumProbability.8;SumAverage.8;SumEntropy.8;SumSquares.8 |
|  | GLSZM(n=127) | GrayLevelNonUniformity.5GrayLevelNonUniformityNormalized.3;GrayLevelVariance.5;HighGrayLevelZoneEmphasis.1;LargeAreaEmphasis.1;LargeAreaHighGrayLevelEmphasis.1;LargeAreaLowGrayLevelEmphasis.1;LowGrayLevelZoneEmphasis.1;SizeZoneNonUniformity.1;SizeZoneNonUniformityNormalized.1;SmallAreaEmphasis.1;SmallAreaHighGrayLevelEmphasis.1;SmallAreaLowGrayLevelEmphasis.1;ZoneEntropy.1;ZonePercentage.1;ZoneVariance.1;GrayLevelNonUniformity.8;GrayLevelNonUniformityNormalized.5;GrayLevelVariance.8;HighGrayLevelZoneEmphasis.2;LargeAreaEmphasis.2;LargeAreaHighGrayLevelEmphasis.2;LargeAreaLowGrayLevelEmphasis.2;LowGrayLevelZoneEmphasis.2;SizeZoneNonUniformity.2;SizeZoneNonUniformityNormalized.2;SmallAreaEmphasis.2;SmallAreaHighGrayLevelEmphasis.2;SmallAreaLowGrayLevelEmphasis.2;ZoneEntropy.2;ZonePercentage.2;ZoneVariance.2;GrayLevelNonUniformity.11;GrayLevelNonUniformityNormalized.7;GrayLevelVariance.11;HighGrayLevelZoneEmphasis.3;LargeAreaEmphasis.3;LargeAreaHighGrayLevelEmphasis.3;LargeAreaLowGrayLevelEmphasis.3;LowGrayLevelZoneEmphasis.3;SizeZoneNonUniformity.3;SizeZoneNonUniformityNormalized.3;SmallAreaEmphasis.3;SmallAreaHighGrayLevelEmphasis.3;SmallAreaLowGrayLevelEmphasis.3;ZoneEntropy.3;ZonePercentage.3;ZoneVariance.3;GrayLevelNonUniformity.14;GrayLevelNonUniformityNormalized.9;GrayLevelVariance.14;HighGrayLevelZoneEmphasis.4;LargeAreaEmphasis.4;LargeAreaHighGrayLevelEmphasis.4;LargeAreaLowGrayLevelEmphasis.4;LowGrayLevelZoneEmphasis.4;SizeZoneNonUniformity.4;SizeZoneNonUniformityNormalized.4;SmallAreaEmphasis.4;SmallAreaHighGrayLevelEmphasis.4;SmallAreaLowGrayLevelEmphasis.4;ZoneEntropy.4;ZonePercentage.4;ZoneVariance.4;GrayLevelNonUniformity.17;GrayLevelNonUniformityNormalized.1;GrayLevelVariance.17;HighGrayLevelZoneEmphasis.5;LargeAreaEmphasis.5;LargeAreaHighGrayLevelEmphasis.5;LargeAreaLowGrayLevelEmphasis.5;LowGrayLevelZoneEmphasis.5;SizeZoneNonUniformity.5;SizeZoneNonUniformityNormalized.5;SmallAreaEmphasis.5;SmallAreaHighGrayLevelEmphasis.5;SmallAreaLowGrayLevelEmphasis.5;ZoneEntropy.5;ZonePercentage.5;ZoneVariance.5;GrayLevelNonUniformity.20;GrayLevelNonUniformityNormalized.13;GrayLevelVariance.20;HighGrayLevelZoneEmphasis.6;LargeAreaEmphasis.6;LargeAreaHighGrayLevelEmphasis.6;LargeAreaLowGrayLevelEmphasis.6;LowGrayLevelZoneEmphasis.6;SizeZoneNonUniformity.6;SizeZoneNonUniformityNormalized.6;SmallAreaEmphasis.6;SmallAreaHighGrayLevelEmphasis.6;SmallAreaLowGrayLevelEmphasis.6;ZoneEntropy.6;ZonePercentage.6;ZoneVariance.6;GrayLevelNonUniformityNormalized.15;GrayLevelVariance.23;HighGrayLevelZoneEmphasis.7;LargeAreaEmphasis.7;LargeAreaHighGrayLevelEmphasis.7;LargeAreaLowGrayLevelEmphasis.7;LowGrayLevelZoneEmphasis.7;SizeZoneNonUniformity.7;SizeZoneNonUniformityNormalized.7;SmallAreaEmphasis.7;SmallAreaHighGrayLevelEmphasis.7;SmallAreaLowGrayLevelEmphasis.7;ZoneEntropy.7;ZonePercentage.7;ZoneVariance.7;GrayLevelNonUniformity.26;GrayLevelNonUniformityNormalized.17;GrayLevelVariance.26;HighGrayLevelZoneEmphasis.8;LargeAreaEmphasis.8;LargeAreaHighGrayLevelEmphasis.8;LargeAreaLowGrayLevelEmphasis.8;LowGrayLevelZoneEmphasis.8;SizeZoneNonUniformity.8;SizeZoneNonUniformityNormalized.8;SmallAreaEmphasis.8;SmallAreaHighGrayLevelEmphasis.8;SmallAreaLowGrayLevelEmphasis.8;ZoneEntropy.8;ZonePercentage.8;ZoneVariance.8 |
|  | GLRLM(n=129) | GrayLevelNonUniformity.4;GrayLevelNonUniformityNormalized.2;GrayLevelVariance.4;HighGrayLevelRunEmphasis.1;LongRunEmphasis.1;LongRunHighGrayLevelEmphasis.1;LongRunLowGrayLevelEmphasis.1;LowGrayLevelRunEmphasis.1;RunEntropy.1;RunLengthNonUniformity.1;RunLengthNonUniformityNormalized.1;RunPercentage.1;RunVariance.1;ShortRunEmphasis.1;ShortRunHighGrayLevelEmphasis.1;ShortRunLowGrayLevelEmphasis.1;GrayLevelNonUniformity.7;GrayLevelNonUniformityNormalized.4;GrayLevelVariance.7;HighGrayLevelRunEmphasis.2;LongRunEmphasis.2;LongRunHighGrayLevelEmphasis.2;LongRunLowGrayLevelEmphasis.2;LowGrayLevelRunEmphasis.2;RunEntropy.2;RunLengthNonUniformity.2;RunLengthNonUniformityNormalized.2;RunPercentage.2;RunVariance.2;ShortRunEmphasis.2;ShortRunHighGrayLevelEmphasis.2;ShortRunLowGrayLevelEmphasis.2;GrayLevelNonUniformity.10;GrayLevelNonUniformityNormalized.6;GrayLevelVariance.10;HighGrayLevelRunEmphasis.3;LongRunEmphasis.3;LongRunHighGrayLevelEmphasis.3;LongRunLowGrayLevelEmphasis.3;LowGrayLevelRunEmphasis.3;RunEntropy.3;RunLengthNonUniformity.3;RunLengthNonUniformityNormalized.3;RunPercentage.3;RunVariance.3;ShortRunEmphasis.3;ShortRunHighGrayLevelEmphasis.3;ShortRunLowGrayLevelEmphasis.3;GrayLevelNonUniformity.13;GrayLevelNonUniformityNormalized.8;GrayLevelVariance.13;HighGrayLevelRunEmphasis.4;LongRunEmphasis.4;LongRunHighGrayLevelEmphasis.4;LongRunLowGrayLevelEmphasis.4;LowGrayLevelRunEmphasis.4;RunEntropy.4;RunLengthNonUniformity.4;RunLengthNonUniformityNormalized.4;RunPercentage.4;RunVariance.4;ShortRunEmphasis.4;ShortRunHighGrayLevelEmphasis.4;ShortRunLowGrayLevelEmphasis.4;GrayLevelNonUniformity.16;GrayLevelNonUniformityNormalized.10;GrayLevelVariance.16;HighGrayLevelRunEmphasis.5;LongRunEmphasis.5;LongRunHighGrayLevelEmphasis.5;LongRunLowGrayLevelEmphasis.5;LowGrayLevelRunEmphasis.5;RunEntropy.5;RunLengthNonUniformity.5;RunLengthNonUniformityNormalized.5;RunPercentage.5;RunVariance.5;ShortRunEmphasis.5;ShortRunHighGrayLevelEmphasis.5;ShortRunLowGrayLevelEmphasis.5;GrayLevelNonUniformity.19;GrayLevelNonUniformityNormalized.12;GrayLevelVariance.19;HighGrayLevelRunEmphasis.6;LongRunEmphasis.6;LongRunHighGrayLevelEmphasis.6;LongRunLowGrayLevelEmphasis.6;LowGrayLevelRunEmphasis.6;RunEntropy.6;RunLengthNonUniformity.6;RunLengthNonUniformityNormalized.6;RunPercentage.6;RunVariance.6;ShortRunEmphasis.6;ShortRunHighGrayLevelEmphasis.6;ShortRunLowGrayLevelEmphasis.6;GrayLevelNonUniformity.22;GrayLevelNonUniformityNormalized.14;GrayLevelVariance.22;HighGrayLevelRunEmphasis.7;LongRunEmphasis.7;LongRunHighGrayLevelEmphasis.7;LongRunLowGrayLevelEmphasis.7;LowGrayLevelRunEmphasis.7;RunEntropy.7;RunLengthNonUniformity.7;RunLengthNonUniformityNormalized.7;RunPercentage.7;RunVariance.7;ShortRunEmphasis.7;ShortRunHighGrayLevelEmphasis.7;ShortRunLowGrayLevelEmphasis.7;GrayLevelNonUniformity.23;GrayLevelNonUniformity.25;GrayLevelNonUniformityNormalized.16;GrayLevelVariance.25;HighGrayLevelRunEmphasis.8;LongRunEmphasis.8;LongRunHighGrayLevelEmphasis.8;LongRunLowGrayLevelEmphasis.8;LowGrayLevelRunEmphasis.8;RunEntropy.8;RunLengthNonUniformity.8;RunLengthNonUniformityNormalized.8;RunPercentage.8;RunVariance.8;ShortRunEmphasis.8;ShortRunHighGrayLevelEmphasis.8;ShortRunLowGrayLevelEmphasis.8 |
|  | GLDM(n=98) | DependenceEntropy.2;DependenceNonUniformity.2;DependenceNonUniformityNormalized.2;DependenceVariance.2;GrayLevelNonUniformity.6;GrayLevelVariance.6;HighGrayLevelEmphasis.2;LargeDependenceEmphasis.2;LargeDependenceHighGrayLevelEmphasis.2;LargeDependenceLowGrayLevelEmphasis.2;LowGrayLevelEmphasis.2;SmallDependenceEmphasis.2;SmallDependenceHighGrayLevelEmphasis.2;SmallDependenceLowGrayLevelEmphasis.2;DependenceEntropy.3;DependenceNonUniformity.3;DependenceNonUniformityNormalized.3;DependenceVariance.3;GrayLevelNonUniformity.9;GrayLevelVariance.9;HighGrayLevelEmphasis.3;LargeDependenceEmphasis.3;LargeDependenceHighGrayLevelEmphasis.3;LargeDependenceLowGrayLevelEmphasis.3;LowGrayLevelEmphasis.3;SmallDependenceEmphasis.3;SmallDependenceHighGrayLevelEmphasis.3;SmallDependenceLowGrayLevelEmphasis.3;DependenceEntropy.4;DependenceNonUniformity.4;DependenceNonUniformityNormalized.4;DependenceVariance.4;GrayLevelNonUniformity.12;GrayLevelVariance.12;HighGrayLevelEmphasis.4;LargeDependenceEmphasis.4;LargeDependenceHighGrayLevelEmphasis.4;LargeDependenceLowGrayLevelEmphasis.4;LowGrayLevelEmphasis.4;SmallDependenceEmphasis.4;SmallDependenceHighGrayLevelEmphasis.4;SmallDependenceLowGrayLevelEmphasis.4;DependenceEntropy.5;DependenceNonUniformity.5;DependenceNonUniformityNormalized.5;DependenceVariance.5;GrayLevelNonUniformity.15;GrayLevelVariance.15;HighGrayLevelEmphasis.5;LargeDependenceEmphasis.5;LargeDependenceHighGrayLevelEmphasis.5;LargeDependenceLowGrayLevelEmphasis.5;LowGrayLevelEmphasis.5;SmallDependenceEmphasis.5;SmallDependenceHighGrayLevelEmphasis.5;SmallDependenceLowGrayLevelEmphasis.5;DependenceEntropy.6;DependenceNonUniformity.6;DependenceNonUniformityNormalized.6;DependenceVariance.6;GrayLevelNonUniformity.18;GrayLevelVariance.18;HighGrayLevelEmphasis.6;LargeDependenceEmphasis.6;LargeDependenceHighGrayLevelEmphasis.6;LargeDependenceLowGrayLevelEmphasis.6;LowGrayLevelEmphasis.6;SmallDependenceEmphasis.6;SmallDependenceHighGrayLevelEmphasis.6;SmallDependenceLowGrayLevelEmphasis.6;DependenceEntropy.7;DependenceNonUniformity.7;DependenceNonUniformityNormalized.7;DependenceVariance.7;GrayLevelNonUniformity.21;GrayLevelVariance.21;HighGrayLevelEmphasis.7;LargeDependenceEmphasis.7;LargeDependenceHighGrayLevelEmphasis.7;LargeDependenceLowGrayLevelEmphasis.7;LowGrayLevelEmphasis.7;SmallDependenceEmphasis.7;SmallDependenceHighGrayLevelEmphasis.7;SmallDependenceLowGrayLevelEmphasis.7;DependenceEntropy.8;DependenceNonUniformity.8;DependenceNonUniformityNormalized.8;DependenceVariance.8;GrayLevelNonUniformity.24;GrayLevelVariance.24;HighGrayLevelEmphasis.8;LargeDependenceEmphasis.8;LargeDependenceHighGrayLevelEmphasis.8;LargeDependenceLowGrayLevelEmphasis.8;LowGrayLevelEmphasis.8;SmallDependenceEmphasis.8;SmallDependenceHighGrayLevelEmphasis.8;SmallDependenceLowGrayLevelEmphasis.8 |
|  | NGTDM(n=40) | Busyness.1;Coarseness.1;Complexity.1;Contrast.3;Strength.1;Busyness.2;Coarseness.2;Complexity.2;Contrast.5;Strength.2;Busyness.3;Coarseness.3;Complexity.3;Contrast.7;Strength.3;Busyness.4;Coarseness.4;Complexity.4;Contrast.9;Strength.4;Busyness.5;Coarseness.5;Complexity.5;Contrast.11;Strength.5;Busyness.6;Coarseness.6;Complexity.6;Contrast.13;trength.6;Busyness.7;Coarseness.7;Complexity.7;Contrast.15;Strength.7;Busyness.8;Coarseness.8;Complexity.8;Contrast.17;Strength.8 |

**Appendix E4 ML classifier** **hyperparameters**

RF is essentially a meta-estimator comprising multiple decision trees and makes predictions by ensemble learning method. The optimal hyper-parameters were tuned as follow: the tree n_estimators=100, the tree max_depth=10, the max_festures= auto, min_samples_split=2, min_samples_leaf=1 and min_impurity_decrease=0.

LR is a supervised machine learning algorithm mainly used for classification tasks where the goal is to predict the probability that an instance of belonging to a given class or not. It is a kind of statistical algorithm, which analyze the relationship between a set of independent variables and the dependent binary variables. The optimal hyper-parameters were tuned as follow: the penalty=12, dual=false, tol=0.0001, C=1, fit_intercept= true, intercept_scaling=1, solver=lbfgs, max_iter=100, max_iter=100, multi_class=auto, verbose=0, warm_start= false, class_weight=none, l1_ratio=0.

SVM is a supervised machine learning model that uses classification algorithms for two-group classification problems. The optimal hyper-parameters were tuned as follow: C=1, kernel=rbf, degree=3, gamma=scale, coef0=0, shrinking=true, probability=false, tol=0.001, cache_size=200, class_weight=none, verbose= false, max_iter=-1, decision_function_shape=ovr, break_ties=false.

SGD is an iterative method often used for machine learning, optimizing the gradient descent during each search once a random weight vector is picked. The optimal hyper-parameters were tuned as follow: loss=hinge, penalty=l2, alpha=0.0001, l1_ratio=0.15, fit_interception=ture, verbose=0,epsilon=0.1, learning_rate=optimizer, eta0=0, max_iter=1000, tol=0.001, shuffle=true, early_stopp=false, class_weight=none, warm_start=false, average=false.

MLP is a type of artificial neural network that consists of multiple layers of interconnected nodes, or neurons. Our MLP model consists of two hidden layers, with 100 and 50 neurons respectively, utilizing Rectified Linear Units (ReLU) as the activation function to extract higher-level representations of input features. For optimization, we opted for the Adam optimizer for parameter optimization, while setting the L2 regularization parameter alpha to 0.0001. We constructed a MLP model with robust generalization capabilities and stability for LVI classification tasks, employing strategies such as constant learning rate policy, a maximum of 200 iterations, batch size 'auto', and shuffling of data prior to training.

**Appendix E5 Shukun Medical Research Platform**

Shukun is a world leading intelligent healthcare technology platform that provides intelligent products and innovation solutions to the healthcare industry. Shukun is a disease-centric, comprehensive, end-to-end AI medical image solutions that provides digital doctors for smart surgery. Shukun relies on its own powerful artificial intelligence scientific research platform and cloud platform, collaborates with scientific research institutions and medical institutions to realize the intelligent internet medical model of human-computer collaboration, and develop big data artificial intelligence cross-regional, interdisciplinary.

**Figure legends**

**Fig. S1** Semi-automated 3D whole tumor segmentation on the sagittal C+T1W using 3D slicer via segment editor project.


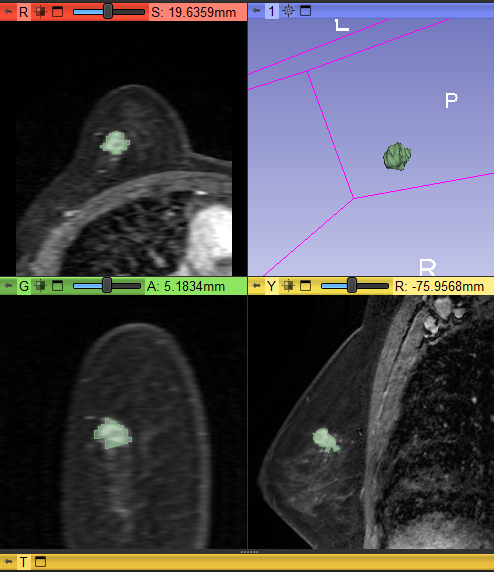


**Fig. S2** (A) After Pearson correlation analysis, the features with highly pairwise correlations at the level of |r|≥ 0.9 were eliminated, and 282 features remained. The thermodynamic chart shows the correlation of those features. (B) Determine the lambda parameter that makes the best accuracy score for the LASSO regression model based on 5-fold cross-validation. (C) The convergence map of variable coefficients in LASSO model depicts the change paths for each feature. The perpendicular corresponds to the most useful radiomic features with non-zero coefficients obtained under optimal lambda value.


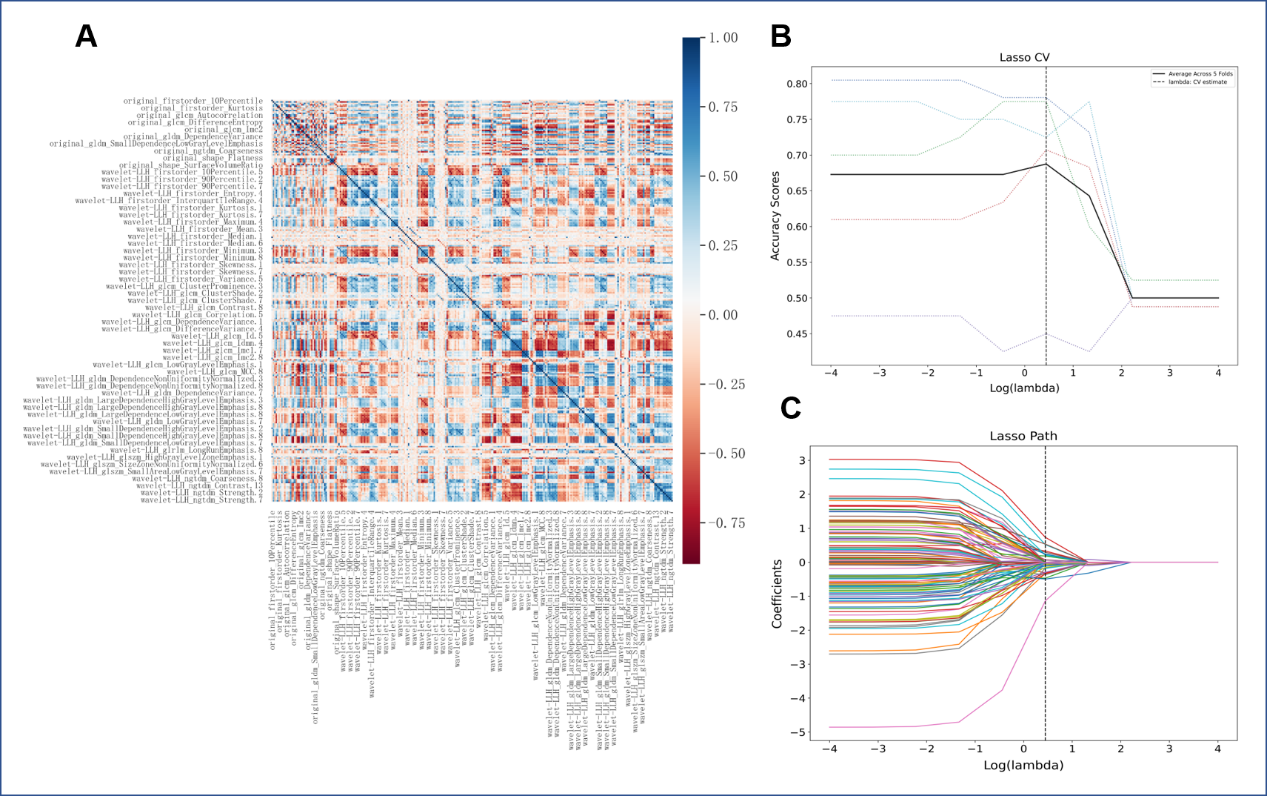


**Fig. S3** The ROC curves of clinicoradiological models based on five classifiers in (A) the training cohorts and (B) the validation cohorts.


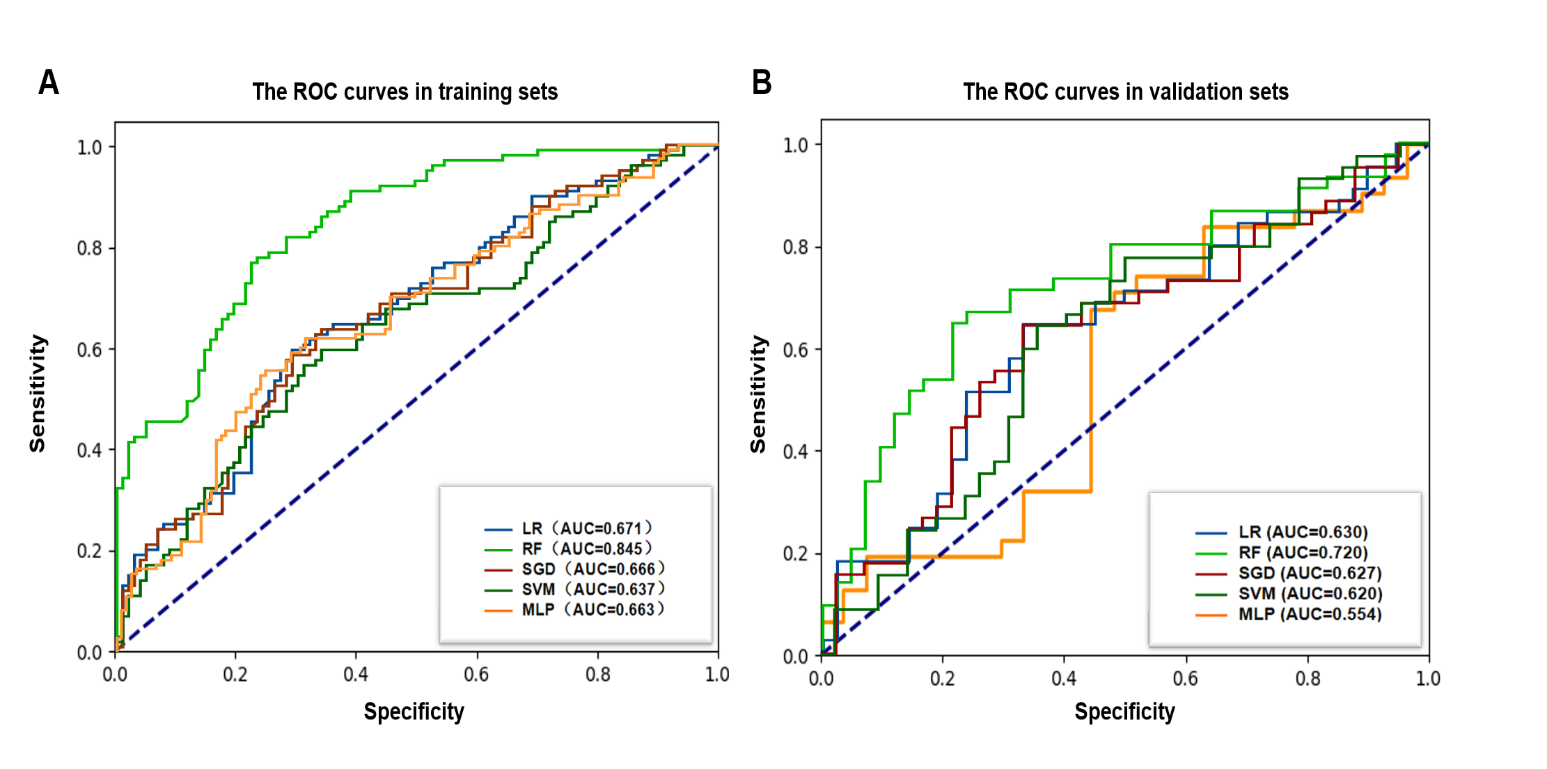


**Table S1** Diagnostic performance of models based on clinicoradiological variables applied to 4 different ML algorithms and MLP

| Models | AUC | 95% CI | | Sensitivity | Specificity | Accuracy | PPV | NPV |
| --- | --- | --- | --- | --- | --- | --- | --- | --- |
|  |  | Lower | Upper |  |  |  |  |  |
| LR |  |  |  |  |  |  |  |  |
| Training set | 0.671 | 0.597 | 0.745 | 0.596 | 0.709 | 0.654 | 0.663 | 0.646 |
| Validation set | 0.630 | 0.511 | 0.749 | 0.667 | 0.644 | 0.655 | 0.636 | 0.674 |
| RF |  |  |  |  |  |  |  |  |
| Training set | 0.845 | 0.793 | 0.897 | 0.778 | 0.767 | 0.772 | 0.762 | 0.782 |
| Validation set | 0.720 | 0.611 | 0.830 | 0.762 | 0.667 | 0.713 | 0.681 | 0.750 |
| SGD |  |  |  |  |  |  |  |  |
| Training set | 0.666 | 0.592 | 0.740 | 0.636 | 0.660 | 0.649 | 0.643 | 0.654 |
| Validation set | 0.627 | 0.507 | 0.746 | 0.714 | 0.556 | 0.632 | 0.600 | 0.676 |
| SVM |  |  |  |  |  |  |  |  |
| Training set | 0.637 | 0.560 | 0.713 | 0.596 | 0.660 | 0.629 | 0.628 | 0.630 |
| Validation set | 0.620 | 0.499 | 0.740 | 0.643 | 0.600 | 0.621 | 0.600 | 0.643 |
| MLP |  |  |  |  |  |  |  |  |
| Training set | 0.663 | 0.592 | 0.732 | 0.736 | 0.438 | 0.580 | 0.544 | 0.646 |
| Validation set | 0.554 | 0.410 | 0.711 | 0.742 | 0.370 | 0.569 | 0.575 | 0.556 |

Abbreviations: AUC, area under the ROC curves; CI, confidence interval; PPV, positive prediction value; NPV, negative prediction value; LR, logistic regression; RF, random forest; SGD, stochastic gradient descent; SVM, support vector machine; MLP, Multilayer Perceptron

*The optimal predictive performance in the validation set was observed in the RF model

**【Noise added in radiomics Models】**

**Logistic Regression**

|  | AUC | APS | ACC | SEN | PPV | SPE | NPV | FPR | FNR | FDR | F1 | Brier |
| --- | --- | --- | --- | --- | --- | --- | --- | --- | --- | --- | --- | --- |
| Training Set | 0.86 | 0.89 | 0.81 | 0.65 | 0.92 | 0.95 | 0.75 | 0.05 | 0.35 | 0.08 | 0.76 | 0.14 |
| Validation Set | 0.85 | 0.89 | 0.79 | 0.74 | 0.85 | 0.85 | 0.74 | 0.15 | 0.26 | 0.15 | 0.79 | 0.16 |

**RF**

|  | AUC | APS | ACC | SEN | PPV | SPE | NPV | FPR | FNR | FDR | F1 | Brier |
| --- | --- | --- | --- | --- | --- | --- | --- | --- | --- | --- | --- | --- |
| Training Set | 1.00 | 1.00 | 1.00 | 1.00 | 1.00 | 1.00 | 1.00 | 0.00 | 0.00 | 0.00 | 1.00 | 0.02 |
| Validation Set | 0.87 | 0.91 | 0.81 | 0.74 | 0.88 | 0.89 | 0.75 | 0.11 | 0.26 | 0.12 | 0.81 | 0.14 |

**SVM**

|  | AUC | APS | ACC | SEN | PPV | SPE | NPV | FPR | FNR | FDR | F1 | Brier |
| --- | --- | --- | --- | --- | --- | --- | --- | --- | --- | --- | --- | --- |
| Training Set | 0.50 | 0.47 | 0.53 | 0.01 | 1.00 | 1.00 | 0.53 | 0.00 | 0.99 | 0.00 | 0.02 | 0.25 |
| Validation Set | 0.50 | 0.53 | 0.47 | 0.00 | 0.00 | 1.00 | 0.47 | 0.00 | 1.00 | 1.00 | 0.00 | 0.25 |

**SGD**

|  | AUC | APS | ACC | SEN | PPV | SPE | NPV | FPR | FNR | FDR | F1 | Brier |
| --- | --- | --- | --- | --- | --- | --- | --- | --- | --- | --- | --- | --- |
| Training Set | 0.56 | 0.51 | 0.55 | 0.72 | 0.52 | 0.40 | 0.61 | 0.60 | 0.28 | 0.48 | 0.61 | 0.45 |
| Validation Set | 0.56 | 0.57 | 0.57 | 0.71 | 0.58 | 0.41 | 0.55 | 0.59 | 0.29 | 0.42 | 0.64 | 0.43 |

**MLP**

|  | AUC | APS | ACC | SEN | PPV | SPE | NPV | FPR | FNR | FDR | F1 | Brier |
| --- | --- | --- | --- | --- | --- | --- | --- | --- | --- | --- | --- | --- |
| Training Set | 0.91 | 0.93 | 0.81 | 0.86 | 0.77 | 0.77 | 0.86 | 0.23 | 0.14 | 0.23 | 0.82 | 0.14 |
| Validation Set | 0.81 | 0.88 | 0.78 | 0.71 | 0.85 | 0.85 | 0.72 | 0.15 | 0.29 | 0.15 | 0.77 | 0.20 |

**【Robustness of the methods】**

**Omics**

| LR |  | AUC | SEN | SPE | ACC | F1 | PPV | NPV | FNR | FDR | FPR |
| --- | --- | --- | --- | --- | --- | --- | --- | --- | --- | --- | --- |
| Fold | 1 | 0.917 | 0.722 | 0.957 | 0.845 | 0.828 | 0.959 | 0.800 | 0.241 | 0.091 | 0.073 |
|  | 2 | 0.915 | 0.754 | 0.968 | 0.857 | 0.838 | 0.951 | 0.805 | 0.215 | 0.101 | 0.085 |
|  | 3 | 0.903 | 0.712 | 0.976 | 0.858 | 0.837 | 0.967 | 0.782 | 0.263 | 0.033 | 0.024 |
|  | 4 | 0.918 | 0.785 | 0.970 | 0.875 | 0.857 | 0.957 | 0.820 | 0.203 | 0.074 | 0.060 |
|  | 5 | 0.886 | 0.712 | 0.984 | 0.846 | 0.820 | 0.970 | 0.764 | 0.279 | 0.050 | 0.036 |
| Mean |  | 0.908 | 0.737 | 0.971 | 0.856 | 0.836 | 0.961 | 0.794 | 0.240 | 0.070 | 0.056 |
| Std |  | 0.014 | 0.032 | 0.010 | 0.012 | 0.014 | 0.008 | 0.022 | 0.032 | 0.028 | 0.025 |

| RF |  | AUC | SEN | SPE | ACC | F1 | PPV | NPV | FNR | FDR | FPR |
| --- | --- | --- | --- | --- | --- | --- | --- | --- | --- | --- | --- |
| Fold | 1 | 0.981 | 0.998 | 0.995 | 0.997 | 0.996 | 0.995 | 0.997 | 0.011 | 0.032 | 0.041 |
|  | 2 | 0.962 | 0.929 | 0.932 | 0.912 | 0.955 | 0.925 | 0.899 | 0.101 | 0.067 | 0.063 |
|  | 3 | 0.958 | 0.779 | 0.951 | 0.873 | 0.918 | 0.938 | 0.798 | 0.151 | 0.079 | 0.059 |
|  | 4 | 0.971 | 0.897 | 0.923 | 0.892 | 0.842 | 0.921 | 0.867 | 0.202 | 0.089 | 0.078 |
|  | 5 | 0.995 | 0.793 | 0.911 | 0.883 | 0.821 | 0.902 | 0.889 | 0.142 | 0.059 | 0.047 |
| Mean |  | 0.973 | 0.879 | 0.942 | 0.911 | 0.906 | 0.936 | 0.890 | 0.121 | 0.065 | 0.058 |
| Std |  | 0.015 | 0.093 | 0.033 | 0.050 | 0.074 | 0.035 | 0.072 | 0.071 | 0.022 | 0.014 |

| SGD |  | AUC | SEN | SPE | ACC | F1 | PPV | NPV | FNR | FDR | FPR |
| --- | --- | --- | --- | --- | --- | --- | --- | --- | --- | --- | --- |
| Fold | 1 | 0.918 | 0.702 | 0.973 | 0.853 | 0.822 | 0.951 | 0.785 | 0.266 | 0.049 | 0.037 |
|  | 2 | 0.917 | 0.766 | 0.995 | 0.854 | 0.852 | 0.991 | 0.812 | 0.165 | 0.132 | 0.122 |
|  | 3 | 0.905 | 0.703 | 0.976 | 0.846 | 0.815 | 0.962 | 0.743 | 0.288 | 0.034 | 0.024 |
|  | 4 | 0.906 | 0.711 | 0.976 | 0.869 | 0.841 | 0.981 | 0.802 | 0.253 | 0.033 | 0.024 |
|  | 5 | 0.899 | 0.704 | 0.986 | 0.838 | 0.801 | 0.982 | 0.774 | 0.266 | 0.108 | 0.084 |
| Mean |  | 0.909 | 0.717 | 0.981 | 0.852 | 0.826 | 0.973 | 0.783 | 0.248 | 0.071 | 0.058 |
| Std |  | 0.008 | 0.028 | 0.009 | 0.011 | 0.020 | 0.016 | 0.027 | 0.048 | 0.046 | 0.043 |

| SVM |  | AUC | SEN | SPE | ACC | F1 | PPV | NPV | FNR | FDR | FPR |
| --- | --- | --- | --- | --- | --- | --- | --- | --- | --- | --- | --- |
| Fold | 1 | 0.934 | 0.711 | 0.995 | 0.851 | 0.834 | 0.992 | 0.811 | 0.215 | 0.101 | 0.085 |
|  | 2 | 0.922 | 0.722 | 0.984 | 0.863 | 0.821 | 0.979 | 0.773 | 0.228 | 0.062 | 0.049 |
|  | 3 | 0.911 | 0.792 | 0.959 | 0.846 | 0.843 | 0.931 | 0.821 | 0.138 | 0.169 | 0.171 |
|  | 4 | 0.901 | 0.708 | 0.988 | 0.877 | 0.852 | 0.984 | 0.802 | 0.241 | 0.016 | 0.012 |
|  | 5 | 0.871 | 0.702 | 0.977 | 0.845 | 0.812 | 0.981 | 0.738 | 0.266 | 0.079 | 0.06 |
| Mean |  | 0.908 | 0.727 | 0.981 | 0.856 | 0.832 | 0.973 | 0.789 | 0.218 | 0.085 | 0.075 |
| Std |  | 0.024 | 0.037 | 0.014 | 0.014 | 0.016 | 0.024 | 0.034 | 0.048 | 0.056 | 0.060 |

| MLP |  | AUC | SEN | SPE | ACC | F1 | PPV | NPV | FNR | FDR | FPR |
| --- | --- | --- | --- | --- | --- | --- | --- | --- | --- | --- | --- |
| Fold | 1 | 0.937 | 0.818 | 0.712 | 0.781 | 0.821 | 0.786 | 0.811 | 0.235 | 0.061 | 0.058 |
|  | 2 | 0.926 | 0.929 | 0.704 | 0.792 | 0.822 | 0.772 | 0.873 | 0.223 | 0.072 | 0.079 |
|  | 3 | 0.915 | 0.892 | 0.742 | 0.862 | 0.813 | 0.731 | 0.821 | 0.145 | 0.069 | 0.154 |
|  | 4 | 0.921 | 0.914 | 0.682 | 0.779 | 0.802 | 0.684 | 0.942 | 0.231 | 0.066 | 0.112 |
|  | 5 | 0.851 | 0.902 | 0.671 | 0.748 | 0.804 | 0.681 | 0.935 | 0.277 | 0.069 | 0.022 |
| Mean |  | 0.910 | 0.891 | 0.702 | 0.792 | 0.812 | 0.731 | 0.876 | 0.222 | 0.067 | 0.085 |
| Std |  | 0.034 | 0.043 | 0.028 | 0.042 | 0.009 | 0.049 | 0.061 | 0.048 | 0.004 | 0.051 |

**Clinical**

| LR |  | AUC | SEN | SPE | ACC | F1 | PPV | NPV | FNR | FDR | FPR |
| --- | --- | --- | --- | --- | --- | --- | --- | --- | --- | --- | --- |
| Fold | 1 | 0.693 | 0.725 | 0.620 | 0.678 | 0.701 | 0.645 | 0.712 | 0.252 | 0.352 | 0.382 |
|  | 2 | 0.654 | 0.641 | 0.570 | 0.609 | 0.621 | 0.583 | 0.620 | 0.351 | 0.412 | 0.431 |
|  | 3 | 0.613 | 0.552 | 0.670 | 0.628 | 0.592 | 0.605 | 0.631 | 0.419 | 0.391 | 0.332 |
|  | 4 | 0.654 | 0.434 | 0.943 | 0.675 | 0.583 | 0.813 | 0.611 | 0.553 | 0.184 | 0.104 |
|  | 5 | 0.742 | 0.630 | 0.744 | 0.681 | 0.669 | 0.671 | 0.658 | 0.347 | 0.323 | 0.305 |
| Mean |  | 0.671 | 0.596 | 0.709 | 0.654 | 0.633 | 0.663 | 0.646 | 0.384 | 0.332 | 0.311 |
| Std |  | 0.049 | 0.110 | 0.146 | 0.033 | 0.051 | 0.090 | 0.041 | 0.111 | 0.090 | 0.125 |

| RF |  | AUC | SEN | SPE | ACC | F1 | PPV | NPV | FNR | FDR | FPR |
| --- | --- | --- | --- | --- | --- | --- | --- | --- | --- | --- | --- |
| Fold | 1 | 0.773 | 0.752 | 0.698 | 0.754 | 0.512 | 0.703 | 0.755 | 0.499 | 0.472 | 0.431 |
|  | 2 | 0.902 | 0.847 | 0.683 | 0.759 | 0.542 | 0.750 | 0.832 | 0.501 | 0.413 | 0.334 |
|  | 3 | 0.865 | 0.687 | 0.759 | 0.765 | 0.651 | 0.842 | 0.768 | 0.371 | 0.331 | 0.292 |
|  | 4 | 0.814 | 0.743 | 0.798 | 0.852 | 0.484 | 0.754 | 0.856 | 0.598 | 0.384 | 0.253 |
|  | 5 | 0.871 | 0.861 | 0.898 | 0.731 | 0.709 | 0.761 | 0.701 | 0.351 | 0.241 | 0.199 |
| Mean |  | 0.845 | 0.778 | 0.767 | 0.772 | 0.580 | 0.762 | 0.782 | 0.464 | 0.368 | 0.302 |
| Std |  | 0.051 | 0.074 | 0.087 | 0.046 | 0.086 | 0.050 | 0.062 | 0.102 | 0.087 | 0.088 |

| SGD |  | AUC | SEN | SPE | ACC | F1 | PPV | NPV | FNR | FDR | FPR |
| --- | --- | --- | --- | --- | --- | --- | --- | --- | --- | --- | --- |
| Fold | 1 | 0.559 | 0.404 | 0.619 | 0.518 | 0.444 | 0.650 | 0.632 | 0.601 | 0.502 | 0.381 |
|  | 2 | 0.657 | 0.901 | 0.691 | 0.593 | 0.656 | 0.588 | 0.670 | 0.467 | 0.512 | 0.995 |
|  | 3 | 0.618 | 0.631 | 0.554 | 0.674 | 0.567 | 0.552 | 0.614 | 0.365 | 0.446 | 0.479 |
|  | 4 | 0.756 | 0.502 | 0.775 | 0.758 | 0.670 | 0.743 | 0.653 | 0.552 | 0.504 | 0.996 |
|  | 5 | 0.738 | 0.741 | 0.658 | 0.704 | 0.708 | 0.680 | 0.701 | 0.251 | 0.318 | 0.324 |
| Mean |  | 0.666 | 0.636 | 0.659 | 0.649 | 0.609 | 0.643 | 0.654 | 0.447 | 0.456 | 0.635 |
| Std |  | 0.082 | 0.196 | 0.082 | 0.095 | 0.106 | 0.075 | 0.034 | 0.142 | 0.082 | 0.334 |

| SVM |  | AUC | SEN | SPE | ACC | F1 | PPV | NPV | FNR | FDR | FPR |
| --- | --- | --- | --- | --- | --- | --- | --- | --- | --- | --- | --- |
| Fold | 1 | 0.595 | 0.451 | 0.571 | 0.542 | 0.474 | 0.654 | 0.522 | 0.551 | 0.502 | 0.429 |
|  | 2 | 0.702 | 0.652 | 0.714 | 0.683 | 0.666 | 0.683 | 0.683 | 0.352 | 0.318 | 0.291 |
|  | 3 | 0.641 | 0.628 | 0.623 | 0.636 | 0.621 | 0.602 | 0.654 | 0.371 | 0.404 | 0.379 |
|  | 4 | 0.543 | 0.449 | 0.719 | 0.539 | 0.487 | 0.531 | 0.520 | 0.547 | 0.467 | 0.404 |
|  | 5 | 0.704 | 0.802 | 0.702 | 0.744 | 0.718 | 0.672 | 0.751 | 0.201 | 0.330 | 0.402 |
| Mean |  | 0.637 | 0.596 | 0.666 | 0.629 | 0.593 | 0.628 | 0.626 | 0.404 | 0.404 | 0.381 |
| Std |  | 0.070 | 0.149 | 0.066 | 0.089 | 0.109 | 0.063 | 0.102 | 0.148 | 0.081 | 0.053 |

| \| MLP \|  \| AUC \| SEN \| SPE \| ACC \| F1 \| PPV \| NPV \| FNR \| FDR \| FPR \| \| --- \| --- \| --- \| --- \| --- \| --- \| --- \| --- \| --- \| --- \| --- \| --- \| \| Fold \| 1 \| 0.674 \| 0.812 \| 0.371 \| 0.626 \| 0.267 \| 0.425 \| 0.643 \| 0.811 \| 0.570 \| 0.304 \| \|  \| 2 \| 0.671 \| 0.704 \| 0.417 \| 0.57 \| 0.477 \| 0.518 \| 0.628 \| 0.557 \| 0.474 \| 0.303 \| \|  \| 3 \| 0.654 \| 0.694 \| 0.352 \| 0.532 \| 0.225 \| 0.566 \| 0.617 \| 0.862 \| 0.431 \| 0.095 \| \|  \| 4 \| 0.634 \| 0.802 \| 0.381 \| 0.581 \| 0.260 \| 0.625 \| 0.641 \| 0.825 \| 0.383 \| 0.111 \| \|  \| 5 \| 0.682 \| 0.666 \| 0.667 \| 0.59 \| 0.143 \| 0.587 \| 0.702 \| 0.930 \| 0.004 \| 0.003 \| \| Mean \|  \| 0.663 \| 0.736 \| 0.438 \| 0.580 \| 0.274 \| 0.544 \| 0.646 \| 0.797 \| 0.372 \| 0.163 \| \| Std \|  \| 0.019 \| 0.067 \| 0.130 \| 0.034 \| 0.124 \| 0.077 \| 0.033 \| 0.142 \| 0.217 \| 0.135 \| |  |  |  |  |  |  |  |  |  |  |  |
| --- | --- | --- | --- | --- | --- | --- | --- | --- | --- | --- | --- | --- | --- | --- | --- | --- | --- | --- | --- | --- | --- | --- | --- | --- | --- | --- | --- | --- | --- | --- | --- | --- | --- | --- | --- | --- | --- | --- | --- | --- | --- | --- | --- | --- | --- | --- | --- | --- | --- | --- | --- | --- | --- | --- | --- | --- | --- | --- | --- | --- | --- | --- | --- | --- | --- | --- | --- | --- | --- | --- | --- | --- | --- | --- | --- | --- | --- | --- | --- | --- | --- | --- | --- | --- | --- | --- | --- | --- | --- | --- | --- | --- | --- | --- | --- | --- | --- | --- | --- | --- | --- | --- | --- | --- | --- | --- | --- |

**Joint**

| LR |  | AUC | SEN | SPE | ACC | F1 | PPV | NPV | FNR | FDR | FPR |
| --- | --- | --- | --- | --- | --- | --- | --- | --- | --- | --- | --- |
| Fold | 1 | 0.866 | 0.782 | 0.904 | 0.804 | 0.781 | 0.889 | 0.764 | 0.302 | 0.132 | 0.101 |
|  | 2 | 0.909 | 0.853 | 0.928 | 0.883 | 0.868 | 0.898 | 0.866 | 0.151 | 0.114 | 0.103 |
|  | 3 | 0.928 | 0.848 | 0.954 | 0.904 | 0.890 | 0.968 | 0.879 | 0.161 | 0.062 | 0.048 |
|  | 4 | 0.863 | 0.554 | 0.867 | 0.756 | 0.647 | 0.826 | 0.662 | 0.446 | 0.209 | 0.147 |
|  | 5 | 0.978 | 0.954 | 0.956 | 0.958 | 0.952 | 0.959 | 0.958 | 0.052 | 0.050 | 0.050 |
| Mean |  | 0.909 | 0.798 | 0.922 | 0.861 | 0.828 | 0.908 | 0.826 | 0.222 | 0.113 | 0.090 |
| Std |  | 0.048 | 0.150 | 0.037 | 0.081 | 0.118 | 0.058 | 0.115 | 0.153 | 0.064 | 0.042 |

| RF |  | AUC | SEN | SPE | ACC | F1 | PPV | NPV | FNR | FDR | FPR |
| --- | --- | --- | --- | --- | --- | --- | --- | --- | --- | --- | --- |
| Fold | 1 | 0.940 | 0.971 | 0.995 | 0.859 | 0.820 | 0.998 | 0.978 | 0.302 | 0.004 | 0.003 |
|  | 2 | 0.988 | 0.975 | 0.901 | 0.862 | 0.814 | 0.887 | 0.975 | 0.249 | 0.120 | 0.102 |
|  | 3 | 0.957 | 0.884 | 0.945 | 0.914 | 0.889 | 0.947 | 0.873 | 0.161 | 0.055 | 0.050 |
|  | 4 | 0.981 | 0.865 | 0.785 | 0.973 | 0.702 | 0.768 | 0.879 | 0.352 | 0.241 | 0.204 |
|  | 5 | 0.995 | 0.901 | 0.891 | 0.945 | 0.901 | 0.904 | 0.901 | 0.098 | 0.102 | 0.104 |
| Mean |  | 0.972 | 0.919 | 0.903 | 0.911 | 0.825 | 0.901 | 0.921 | 0.232 | 0.104 | 0.093 |
| Std |  | 0.023 | 0.051 | 0.078 | 0.050 | 0.079 | 0.086 | 0.052 | 0.103 | 0.089 | 0.075 |

| SGD |  | AUC | SEN | SPE | ACC | F1 | PPV | NPV | FNR | FDR | FPR |
| --- | --- | --- | --- | --- | --- | --- | --- | --- | --- | --- | --- |
| Fold | 1 | 0.452 | 1.000 | 0.000 | 0.490 | 0.660 | 0.495 | 0.000 | 0.001 | 0.512 | 0.998 |
|  | 2 | 0.645 | 0.999 | 0.001 | 0.471 | 0.650 | 0.489 | 0.001 | 0.445 | 0.212 | 0.140 |
|  | 3 | 0.472 | 1.000 | 0.000 | 0.539 | 0.710 | 0.398 | 0.001 | 0.350 | 0.204 | 0.138 |
|  | 4 | 0.424 | 0.999 | 0.001 | 0.461 | 0.430 | 0.472 | 0.000 | 0.701 | 0.248 | 0.104 |
|  | 5 | 0.506 | 1.000 | 0.000 | 0.490 | 0.670 | 0.595 | 0.000 | 0.003 | 0.504 | 0.997 |
| Mean |  | 0.500 | 1.000 | 0.000 | 0.490 | 0.624 | 0.490 | 0.000 | 0.300 | 0.336 | 0.475 |
| Std |  | 0.086 | 0.001 | 0.001 | 0.030 | 0.111 | 0.070 | 0.001 | 0.301 | 0.158 | 0.477 |

| SVM |  | AUC | SEN | SPE | ACC | F1 | PPV | NPV | FNR | FDR | FPR |
| --- | --- | --- | --- | --- | --- | --- | --- | --- | --- | --- | --- |
| Fold | 1 | 0.951 | 0.745 | 0.957 | 0.851 | 0.473 | 0.944 | 0.852 | 0.553 | 0.500 | 0.431 |
|  | 2 | 0.879 | 0.766 | 0.971 | 0.768 | 0.676 | 0.962 | 0.768 | 0.346 | 0.321 | 0.290 |
|  | 3 | 0.864 | 0.747 | 0.888 | 0.798 | 0.513 | 0.851 | 0.758 | 0.532 | 0.442 | 0.332 |
|  | 4 | 0.901 | 0.648 | 0.872 | 0.870 | 0.466 | 0.953 | 0.750 | 0.550 | 0.502 | 0.453 |
|  | 5 | 0.852 | 0.727 | 0.970 | 0.873 | 0.715 | 0.844 | 0.779 | 0.301 | 0.229 | 0.247 |
| Mean |  | 0.889 | 0.727 | 0.932 | 0.832 | 0.569 | 0.911 | 0.781 | 0.456 | 0.399 | 0.351 |
| Std |  | 0.039 | 0.046 | 0.048 | 0.047 | 0.118 | 0.058 | 0.041 | 0.123 | 0.120 | 0.089 |

| MLP |  | AUC | SEN | SPE | ACC | F1 | PPV | NPV | FNR | FDR | FPR |
| --- | --- | --- | --- | --- | --- | --- | --- | --- | --- | --- | --- |
| Fold | 1 | 0.985 | 0.981 | 0.380 | 0.805 | 0.801 | 0.592 | 0.974 | 0.291 | 0.082 | 0.072 |
|  | 2 | 0.882 | 0.998 | 0.378 | 0.737 | 0.720 | 0.577 | 0.978 | 0.320 | 0.231 | 0.151 |
|  | 3 | 0.978 | 0.985 | 0.341 | 0.585 | 0.639 | 0.655 | 0.965 | 0.246 | 0.447 | 0.566 |
|  | 4 | 0.814 | 0.993 | 0.351 | 0.601 | 0.682 | 0.558 | 0.982 | 0.171 | 0.420 | 0.643 |
|  | 5 | 0.976 | 0.999 | 0.451 | 0.627 | 0.657 | 0.580 | 0.998 | 0.263 | 0.413 | 0.470 |
| Mean |  | 0.927 | 0.991 | 0.380 | 0.671 | 0.700 | 0.592 | 0.979 | 0.258 | 0.319 | 0.380 |
| Std |  | 0.076 | 0.008 | 0.043 | 0.096 | 0.064 | 0.037 | 0.012 | 0.056 | 0.158 | 0.255 |
